# The effect of acupuncture on oxidative stress in animal models of vascular dementia: a systematic review and meta-analysis

**DOI:** 10.1186/s13643-024-02463-x

**Published:** 2024-02-08

**Authors:** Qiong-Nan Bao, Man-Ze Xia, Jing Xiong, Yi-Wei Liu, Ya-Qin Li, Xin-Yue Zhang, Zheng-Hong Chen, Jin Yao, Ke-Xin Wu, Wan-Qi Zhong, Shao-Jun Xu, Zi-Han Yin, Fan-Rong Liang

**Affiliations:** 1 Department of Traditional Chinese Medicine, The First People’s Hospital of Yunnan Province, Kunming, Yunnan China; 2 The Affiliated Hospital of Kunming University of Science and Technology, Kunming, Yunnan China; 3 School of Acu-Mox and Tuina, Chengdu University of Traditional Chinese Medicine, Chengdu, Sichuan China; 4 Department of Rehabilitation, West China Hospital of Sichuan University, Chengdu, Sichuan China

**Keywords:** Acupuncture, Vascular dementia, Oxidative stress, Animal model, Meta-analysis

## Abstract

**Background:**

Growing evidence showed that acupuncture may improve cognitive function by reducing oxidative stress, key to the pathogenesis in vascular dementia (VaD), but this is yet to be systematically analysed. This study aimed to summarize and evaluate the effect of acupuncture on oxidative stress in animal models of VaD.

**Method:**

Eight databases including PubMed, Embase, Web of Science, Cochrane library, CNKI, Wan Fang, CBM, and VIP were searched since their establishment until April 2023, for studies that reported the effect of acupuncture on oxidative stress in VaD animal models. Relevant literature was screened, and information was extracted by two reviewers. The primary outcomes were the levels of oxidative stress indicators. The methodological quality was assessed via the SYRCLE Risk of Bias Tool. Statistical analyses were performed using the RevMan and Stata software.

**Results:**

In total, 22 studies with 747 animals were included. The methodology of most studies had flaws or uncertainties. The meta-analysis indicated that, overall, acupuncture significantly reduced the expression of pro-oxidants including reactive oxygen species (standardized mean differences [SMDs] = -4.29, 95% confidence interval [CI]: -6.26, -2.31), malondialdehyde (SMD = -2.27, 95% CI: -3.07, -1.47), nitric oxide (SMD = -0.85, 95% CI: -1.50, -0.20), and nitric oxide synthase (SMD = -1.01, 95% CI: -1.69, -0.34) and enhanced the levels of anti-oxidants including super oxide dismutase (SMD = 2.80, 95% CI: 1.98, 3.61), glutathione peroxidase (SMD = 1.32, 95% CI: -0.11, 2.76), and catalase (SMD = 1.31, 95% CI: 0.05, 2.58) in VaD animal models. In subgroup analyses, acupuncture showed significant effects on most variables. Only partial modelling methods and treatment duration could interpret the heterogeneity of some outcomes.

**Conclusion:**

Acupuncture may inhibit oxidative stress to improve cognitive deficits in animal models of VaD. Nevertheless, the methodological quality is unsatisfactory. More high-quality research with a rigorous design and further experimental researches and clinical trials are needed to confirm these findings.

**Systematic review registration:**

This study was registered in PROSPERO (CRD42023411720).

**Supplementary Information:**

The online version contains supplementary material available at 10.1186/s13643-024-02463-x.

## Introduction

Vascular dementia (VaD) is the second most common type of dementia ranked behind Alzheimer’s disease. The typical symptoms of VaD are progressive cognitive impairment, behavioural abnormalities, affective disorder, and neurological dysfunction caused by cerebrovascular diseases [1, 2]. Epidemiological findings reveal that there are more than 50 million cases of dementia globally. VaD may account for up to 17 million cases with annual costs of up to 200 billion dollars [3]. The prevalence of VaD rises exponentially with the increased ageing of the population, imposing a financial burden on families and the society [4, 5]. Therefore, it is urgent to find effective treatments for VaD.

The major pathological feature of VaD is chronic cerebral hypoperfusion, which develops because the blood supply to brain tissue is below the physiological threshold for a prolonged period [6, 7]. This condition promotes free radical formation, causing mitochondrial dysfunction, inducing white matter abnormalities, and increasing blood brain barrier permeability [8, 9]. Emerging evidence suggests that oxidative stress also plays an important role in the pathogenesis of VaD [10, 11]. Activated microglia in VaD can generate excessive reactive oxygen species (ROS), which can initiate oxidative stress and lead to neuronal damage and apoptosis, thus resulting in neuropathological changes and brain tissue injury and, ultimately, cognitive decline and behavioural dysfunction [12, 13].

Oxidative stress is an environment where the free radicals override cellular antioxidants in the body [14]. There are two types of oxidant species, pro-oxidants and antioxidants [15]. Pro-oxidant species, including ROS, malondialdehyde (MDA), nitric oxide (NO), nitric oxide synthase (NOS), and chlorine species, enhance the oxidative stress response and promote cell death. Conversely, superoxide dismutase (SOD), glutathione peroxidase (GSH-Px), and catalase (CAT), which are considered antioxidant species, inhibit oxidative stress and exert neuroprotective effects [16]. Studies have shown that the susceptibility to oxidative stress increases and the antioxidative defence decreases in patients with VaD [17]. Thus, treatment protecting against oxidative stress may help improve cognitive function and delay disease progression in patients with VaD.

Acupuncture, as an integral part of traditional Chinese medicine, has been used as an alternative and complementary treatment for multiple neurodegenerative diseases including VaD over the past decades [18, 19]. Multiple randomized controlled trials and systematic reviews demonstrated that acupuncture could improve cognitive function and activities of daily living in patients with VaD [20–22]. Meanwhile, the underlying mechanisms are being explored extensively. A recent systematic review summarized that the main mechanism of acupuncture in the treatment of VaD includes reduced oxidative stress, anti-neuroinflammation, and anti-apoptosis, as well as regulation of synaptic plasticity and neurotransmitters [23]. Although numerous experiments have focused on the antioxidative effects of acupuncture on VaD, [24–26], a systematic review regarding the effect of acupuncture on oxidative stress in VaD is still lacking. Accordingly, this preclinical systematic review aimed to summarize and evaluate the evidence for the effect of acupuncture treatment on oxidative stress in VaD, thus providing a reference for further research.

## Methods

### Protocol registration

This systematic review was reported following the latest Preferred Reporting Items for Systematic Reviews and Meta-Analyses (PRISMA) guidelines [27], The PRISMA checklist is described in Additional file 1. The protocol was preregistered in the International Prospective Register of Systematic Reviews (PROSPERO, CRD42023411720).

### Search strategy

Studies that examined the effects of acupuncture on oxidative stress in animal models of VaD were identified. The following databases were searched, without language restriction, from their inception until 11 April 2023: PubMed, Embase, Web of Science, Cochrane library, CNKI, Wan Fang, CBM, and VIP. The search string included “acupuncture” or “electroacupuncture,” “rat (rats)” or “mouse (mice),” and “vascular dementia.” The specific search strategy is described in Additional file 2. Also, reference lists in the selected articles were reviewed manually to obtain any additional relevant studies.

### Eligibility criteria

#### Inclusion criteria

(1) Type of studies: original full text of animal experiments with at least one separate control group; (2) Subjects: animal models of VaD, without restriction on species, age, sex, or modelling methods; (3) Interventions: treatment group received manual acupuncture (MA) or electroacupuncture (EA); (4) Comparisons: control groups received non-intervention, sham acupuncture, or nimodipine (a drug able to improve cognition in VaD because of neuroprotective and vasoactive effects [28]); (5) Outcome measures: data for the levels of oxidative stress indicators, including oxidants (ROS, MDA, NO, NOS) and anti-oxidants (SOD, GSH-Px, CAT), and Morris Water Maze (MWM) test, consisting of escape latency, platform crossing number, duration in the platform quadrant, and swimming speed, were available in the original article.

#### Exclusion criteria

(1) Reviews, clinical trials, case reports, editorials, or conference abstracts; (2) Studies using in vitro or ex vivo models; treatment group using other therapies or combination with other interventions (such as drug, moxibustion, etc.); (3) Studies comparing the clinical efficacy between different acupoints, different acupuncture methods, or comparing acupuncture with other complementary and alternative therapies.

### Data selection

Two independent reviewers (Y.W. Liu, J. Xiong) screened the titles and abstracts to exclude irrelevant studies using NoteExpress v2.7. Full text assessment was then conducted to identify whether the literature corresponded with the inclusion criteria. Any disagreements were resolved by consulting a third researcher (Z.H.Yin).

### Data extraction

Two reviewers (S.J.Xu, Y.Q.Li) independently extracted the following data from the included studies: study identification features (publication year and the first author’s name); animal model characteristics (including species weight, age, modelling method of VaD, and sampling sites); intervention characteristics (such as type of acupuncture, acupoints, intervention time, and parameters); primary outcome measures (levels of each oxidative stress indicator, including ROS, MDA, NO, NOS, SOD, GSH-Px, and CAT), secondary outcome measures (results of the MWM test). If numerical data were not reported in the text, we extracted data from graphs using the GetData Gragh Digitizer v2.26 software.

### Risk of bias assessment

The risk-of-bias (ROB) of each included study was assessed by two independent reviewers (X.Y.Zhang, Z.H.Chen) using the Systematic Review Center for Laboratory Animal Experimentation (SYRCLE) ROB tool [29]. The tool provides 10 items (random sequence generation, baseline characteristics, allocation concealment, random housing, blinding of participants and personnel, random outcome assessment, blinding of outcome assessment, incomplete outcome data, selective reporting, and other sources of bias) involved in six aspects of bias (selection, performance, detection, attrition, reporting, and other). Each item corresponded to signal questions related to ‘low,’ ‘high,’ and ‘unclear’ ROB. If there was any disagreement, it was resolved by a third reviewer (Z.H.Yin).

### Statistics

Data analyses were performed using the RevMan 5.4 and Stata 17.0 software. The types of all data in the present study were continuous; For oxidative stress indicators and swimming speed of the MWM test, standardized mean differences (SMDs) with 95% confidence intervals (CIs) were reported as effect-size indices owing to the differences in measurement units among studies. For other results of the MWM test, the measurement units were consistent among studies; thus, mean differences (MDs) with 95% CIs were presented. A *P* value < 0.05 was considered statistically significant. Chi-square and *I*^2^ statistics were used for heterogeneity assessment. When the *I*^2^ value was > 50%, which meant significant heterogeneity, a random-effects model was applied, and subgroup analyses were carried out to explore the sources of heterogeneity based on different acupuncture stimulation types, modelling methods, and treatment duration. Publication biases were assessed using funnel plots if 10 or more studies were included in a meta-analysis. Moreover, sensitivity analyses were performed by excluding studies with high ROB in at least one domain to explore the source of heterogeneity and test the reliability of the results of meta-analyses.

## Results

### Study selection

**Fig. 1:**
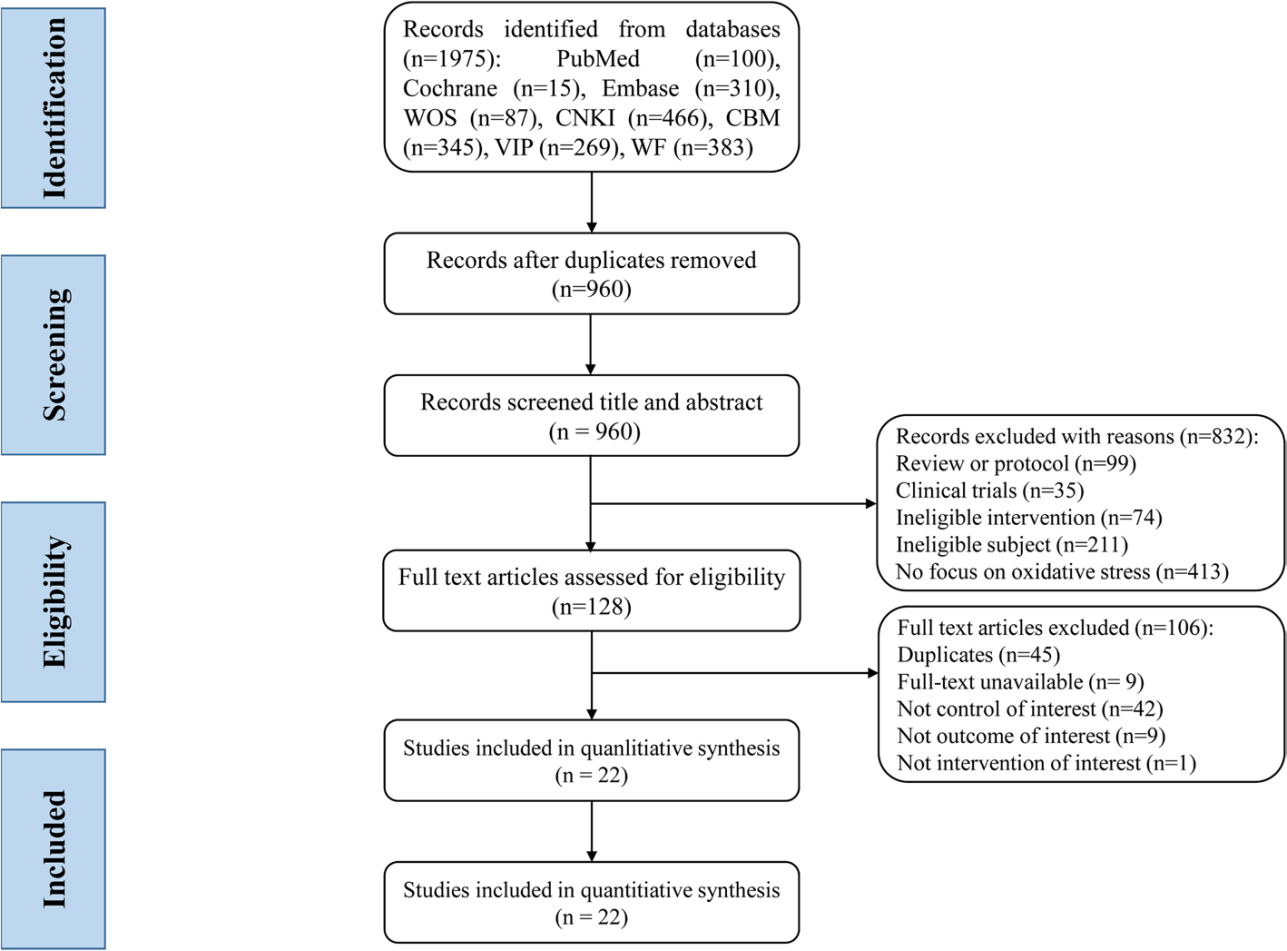
Flowchart of literature selection process and screening results

In total, 1975 relevant studies were retrieved from eight databases, 1015 duplicate records were removed, and 960 articles remained. After screening the titles and abstracts, 832 articles were eliminated and 128 citations entered the full-text reading stage, from which 106 articles were excluded. Ultimately, 22 studies were included in the data syntheses and meta-analysis. The flow diagram of the study selection process is shown in Fig. 1.

### Study characteristics

Twenty-two studies with a total of 747 rats were included; 266 rats were in treatment groups and 481 rats in control groups. These studies were published from 2000 to 2022, of which 15 were in Chinese and 7 in English. The species of experimental animals in the included studies were Sprague Dawley or Wistar rats. Fifteen studies [24–26, 30–41] included only male animals, two [42, 43] included only female, and five [44–48] included equal numbers of each sex. For modelling VaD, eight studies [24–26, 30, 32–35] used the bilateral common carotid artery ligation (AL) method, two [31, 40] used bilateral internal carotid AL method, one [43] used middle cerebral AL method, one [42] used the bilateral common carotid intermittent artery clamp (AC) method, six [36, 41, 45–48] used the 4-vessel occlusion (4-VO) method, and four [37–39, 44] used the thromboembolus method (TM). Only two studies [33, 38] reported treatment by a licensed acupuncturist. No one reported the adverse acupuncture reactions.

In terms of acupuncture stimulation methods, MA was utilized in 12 studies [24–26, 30, 33, 34, 36–39, 44, 45] and EA in 10 studies [31, 32, 35, 40–43, 46–48]. Among them, nine studies [31, 32, 40–43, 46–48] reported the parameters of EA, 4 studies [31, 32, 40, 47] used disperse-dense wave, and 5 studies [41–43, 46, 48] used continuous wave. Nine [31, 32, 40–43, 46–48] and two studies [31, 48] described the frequency and intensity of EA ranging from 1 to 150 Hz and 0.2 mA to 1.5 mA, respectively. All studies described the acupoints, and a total of 19 acupoints were used. Acupoints used most frequently were GV20 (16 times), ST36 (13 times), BL23 (4 times), GV14 (4 times), and SP10 (4 times). GV20–ST36 was the acupoint combination used most often.

In the 22 included studies, seven oxidative stress indicators were reported, including ROS, MDA, NO, NOS, SOD, GSH-Px, and CAT. All studies used the MWM test to measure behavioural changes. For the control group, all studies used non-intervention, and 11 [24–26, 30, 33–35, 37–39, 44] used sham acupuncture in which a non-acupoint was stimulated. Four studies [41, 46–48] used nimodipine. The main features of the included studies are summarized in Table 1.

**Table 1:** Characteristics of the included studies

No	References	language	Animal models						Intervention (treatment group)				Intervention (control group)	License of acupuncturists	Specimen	Oxidative stress indicators	Behavioral tests
			Sources	Species	Sex, number	Age (months), weight	Modelling method	Degree and duration of ischemia	Type	Acupoints	Duration (days)/ frequency (daily)	Electroacupuncture parameters					
1	Li et al., 2021 [26]	Chinese	Laboratory Animal Center, Guangzhou University of TCM	Wistar rat	Male 54	4–5 mo, 300-320 g	BCCA	NR	MA	GV20, ST36	14 d, 1 time	-	Non-intervention group; sham-acupuncture group	NR	Hippocampus	ROS, MDA, SOD	①②
2	Wang et al., 2015 [34]	English	Vital River Laboratory Animal Technology Company	Wistar rat	Male 30	NR, 200-220 g	BCCA	NR	MA	GV20, ST36	14 d, 1 time	-	Non-intervention group; sham-acupuncture group	NR	Hippocampus	ROS	①②③
3	Liu et al., 2004 [44]	Chinese	The 4th Institute of the Chinese People's Liberation Army Academy of Military Medical Sciences	Wistar rat	Female/Male 29	10 mo, 340 ± 40 g	TM	NR	MA	RN17, RN12, RN6, SP10, ST36	21 d, 1 time	-	Non-intervention group; sham-acupuncture group	NR	Hippocampus	MDA, SOD, GSH-Px, NO, NOS	①②④⑤
4	Qiu et al., 2022 [32]	Chinese	Hunan Slack Jingda Experimental Animal Company	Sprague Dawley rat	Male 20	NR, 220-250 g	BCCA	NR	EA	GV20, GV14, BL23	28 d, 1 time	Sparse wave, 10-50HZ	Non-intervention group	NR	Hippocampus	ROS	①④⑤
5	Zhu et al., 2018 [24]	English	Vital River Laboratory Animal Technology Company	Wistar rat	Male 30	NR, 200-220 g	BCCA	NR	MA	GV20, ST36	28 d, 1 time	-	Non-intervention group; sham-acupuncture group	NR	Hippocampus	ROS	①③④⑥
6	Li et al., 2016 [30]	English	Vital River Laboratory Animal Technology Company	Wistar rat	Male 42	NR, 270-320 g	BCCA	NR	MA	GV20, ST36	14 d, 1 time	-	Non-intervention group; sham-acupuncture group	NR	Hippocampus	ROS	①②
7	Du et al., 2018 [33]	English	Vital River Laboratory Animal Technology Company	Wistar rat	Male 30	2.5 mo, NR	BCCA	NR	MA	GV20, ST36	14 d, 1 time	-	Non-intervention group; sham-acupuncture group	Y	Hippocampus	ROS, SOD	①②⑥
8	Yang et al., 2018 [25]	English	Vital River Laboratory Animal Technology Company	Wistar rat	Male 18	2 mo, 280-320 g	BCCA	NR	MA	GV20, ST36	14 d, 1 time	-	Non-intervention group; sham-acupuncture group	NR	Hippocampus	ROS, SOD	①②③⑤
9	Hu et al., 2022 [31]	Chinese	Hunan Slack Jingda Experimental Animal Company	Sprague Dawley rat	Male 18	NR, 220-250 g	BICA	NR	EA	GV20, ST36	14 d, 1 time	Sparse wave, 1-2 Hz, 1.5 mA	Non-intervention group	NR	Hippocampus; serum	SOD, GSH-Px, CAT, MDA, ROS	①②④
10	Cheng et al., 2022 [35]	Chinese	Liaoning Changsheng Biotechnology Company., LTD	Sprague Dawley rat	Male 30	3 mo, 180 ± 20 g	BCCA	NR	EA	ST36, SP6	5 d, 1 time	NR	Non-intervention group; sham-acupuncture group	NR	Hippocampus	ROS, SOD, MDA	①④
11	Song et al., 2018 [42]	Chinese	Shanghai Slack Laboratory Animal Company	Sprague Dawley rat	Female 20	11–15 mo, 370 g	BICOCA	NR	EA	RN4, SP6	20 d, 1 time	Continuous wave, 2 Hz	Non-intervention group	NR	Hippocampus	SOD, MDA	①
12	Li et al., 2017 [36]	Chinese	Laboratory Animal Center, Heilongjiang University of TCM	Sprague Dawley rat	Male 20	3 mo, 280-300 g	4-VO	NR	MA	GV20, GV24, GV14, ST36	16 d, 1 time	-	Non-intervention group	NR	Hippocampus	SOD, MDA	①④
13	Zhang et al., 2014 [37]	English	NR	Wistar rat	Male 30	NR, 300-320 g	TM	NR	MA	CV17, CV12, CV6, ST36, SP10	21 d, 1 time	-	Non-intervention group; sham-acupuncture group	NR	Brain tissues	SOD, MDA	①②④
14	Liu et al., 2013 [38]	English	The 4th Institute of the Chinese People's Liberation Army Academy of Military Medical Sciences	Wistar rat	Male 48	NR, 340 ± 40 g	TM	NR	MA	CV17, CV12, CV6, ST36, SP10	21 d, 1 time	-	Non-intervention group; sham-acupuncture group	Y	Hippocampus	Ref-1	③⑤⑦
15	Ji et al., 2011 [45]	Chinese	Laboratory Animal Center, The Fourth Military Medical University of the People's Liberation Army	Sprague Dawley rat	Female/Male 20	NR, 220-260 g	4-VO	5 min for three times, each with an interval of 1 h	MA	GV20, RN17, RN6, BL17, SP6	30 d, 1 time	-	Non-intervention group; sham-acupuncture group	NR	Hippocampus	SOD, MDA	①④
16	Liu et al., 2010 [39]	Chinese	NR	Wistar rat	Male 90	8 mo, 320-360 g	TM	NR	MA	RN17, RN12, RN6, SP10, ST36	21 d, 1 time	-	Non-intervention group; sham-acupuncture	NR	Hippocampus	GSH, GSSG, Ref-1	①③⑤⑦
17	Yan et al., 2008 [48]	Chinese	Laboratory Animal Center, Guangzhou University of TCM	Sprague Dawley rat	Female/Male 25	NR, 180-220 g	4-VO	5 min for three times, each with an interval of 1 h	EA	GV20, BL17, BL20, BL23	15 d, 1 time	Continuous wave, 150 Hz	Non-intervention group; drug group	NR	Brain tissues	NO	①④
18	Meng et al., 2007 [43]	Chinese	Laboratory Animal Center, Hubei University of TCM	Sprague Dawley rat	Male 67	NR, 200-250 g	MCA	1.5 h	EA	GV20, BL23, Hou San Li (posterolateral knee joint, about 5 mm below the small head of fibula)	15d, 1 time	Continuous wave, 2 Hz	Non-intervention group	NR	Hippocampus	NO, NOS	①④⑤
19	Chen et al., 2006 [47]	Chinese	Laboratory Animal Center, Guangzhou University of TCM	Sprague Dawley rat	1/1 30	NR, 180-200 g	4-VO	5 min for three times, each with an interval of 1 h	EA	GV20, BL23, BL20	15 d, 1 time	Sparse wave,16 Hz	Non-intervention group; drug group	NR	Brain tissues	NO, NOS	①④
20	Wang et al., 2006 [40]	Chinese	Shanghai Laboratory Animal Center, Chinese Academy of Sciences	Sprague Dawley rat	Male 26	NR, 180-200 g	BICA	20 min for two times, each with an interval of 10 min	EA	GV20, 1 point (0.2 cuns far from GV14)	30 d, once 2 days	Sparse wave,15 Hz	Non-intervention group	NR	Brain tissues	NO, SOD	①④
21	Wang et al., 2002 [48]	Chinese	Laboratory Animal Center, Guangzhou University of TCM	Sprague Dawley rat	Female/Male 40	2–3 mo, 200-250 g	4-VO	5 min for three times, each with an interval of 1 h	EA	GV20, GV14	30 d, 1 time	Continuous wave, 150 Hz, 0.2 mA	Non-intervention group; drug group	NR	Brain tissues	NO, NOS, MDA, SOD, GSH-PX	①④
22	Lai et al., 2000 [41]	Chinese	Laboratory Animal Center, Guangzhou University of TCM	Sprague Dawley rat	Male 30	NR, 200-250 g	4-VO	5 min for three times, each with an interval of 1 h	EA	GV20, GV14	10 d, 1 time	Continuous wave, 150 Hz	Non-intervention group; drug group	NR	Brain tissues	MDA, SOD	

### Risk of bias

**Fig. 2:**
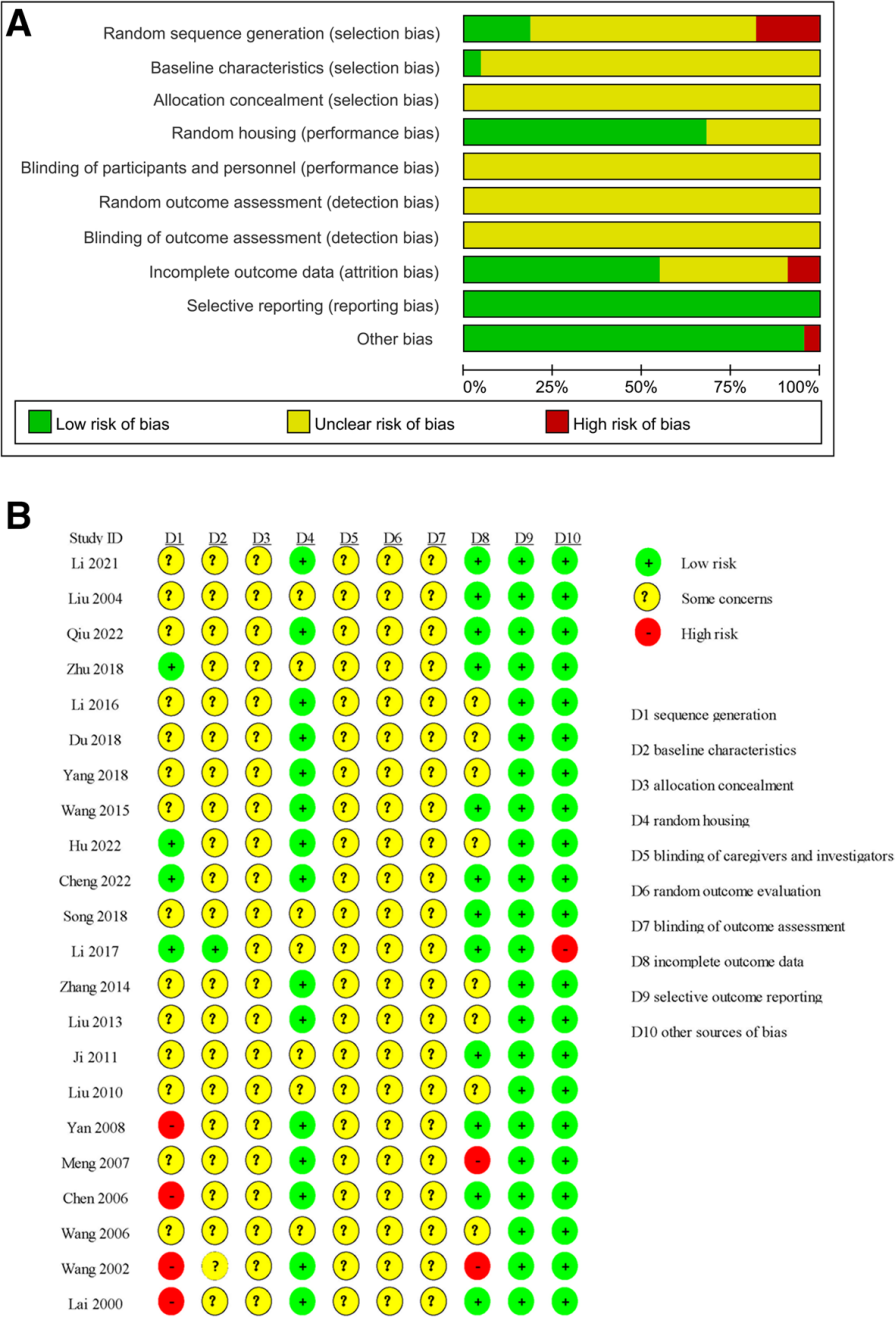
**A** Risk of bias graph **B** Risk of bias summary

Among the 22 included studies, four [31, 32, 35, 36] reported the animals were randomized by using appropriate methods such as a random number table or computer-generated random numbers, whereas four studies [41, 46–48] reported an inappropriate approach for sequence generation, such as by sex or weight of animals. The remaining 14 studies [24–26, 30, 33, 34, 37–40, 42–45] just mentioned “randomized” without providing detailed information. Only one study [36] clarified that there was no statistical difference in baseline data. Almost all the included studies failed to give the specific allocation concealment. Fifteen studies [25, 26, 30–35, 37, 38, 41, 43, 46–48] described animal housing conditions. The blinding of caregivers and investigators was considered not applicable for acupuncture treatment. No study mentioned randomized outcome evaluation or blinded assessment of outcome. In terms of incomplete outcome data, two studies [43, 48] selected a portion of the experimental animals for analysis, and eight studies [25, 30, 31, 33, 37–40] did not specify the number of animals included in the analysis. All studies reported the literature data completely. One study [36] may have other sources of bias because dead animals were replaced with new ones during modelling or feeding treatment. The detailed results are displayed in Fig. 2A and B.

### Meta-analysis

#### ROS

**Fig. 3:**
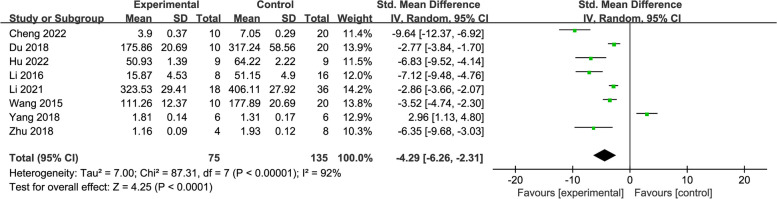
Forest plot of ROS for acupuncture vs. control

Eight studies [24–26, 30, 31, 33–35] reported ROS as an outcome, with 210 rats in total. Meta-analysis results indicated that, overall, acupuncture significantly lowered ROS levels when compared to the control (SMD = -4.29, 95% CI: -6.26, -2.31) (Fig. 3).

Because of the considerable heterogeneity (*I*^2^ = 92%), we further conducted subgroup analyses based on different acupuncture stimulation types and treatment duration. The results suggested that both MA (SMD = -3.56, 95% CI: -5.44, -1.68) and EA (SMD = -9.64, 95% CI: -12.37, -6.92) and treatment durations ≤ 15 days (SMD = -4.05, 95% CI: -6.14, -1.96) and > 15 days (SMD = -6.35, 95% CI: -9.68, -3.03) could significantly reduce ROS levels compared to the control. No subgroup could reduce the heterogeneity (*I*^2^ = 91%; *I*^2^ = 93%) (see Additional file 3).

#### MDA

**Fig. 4:**
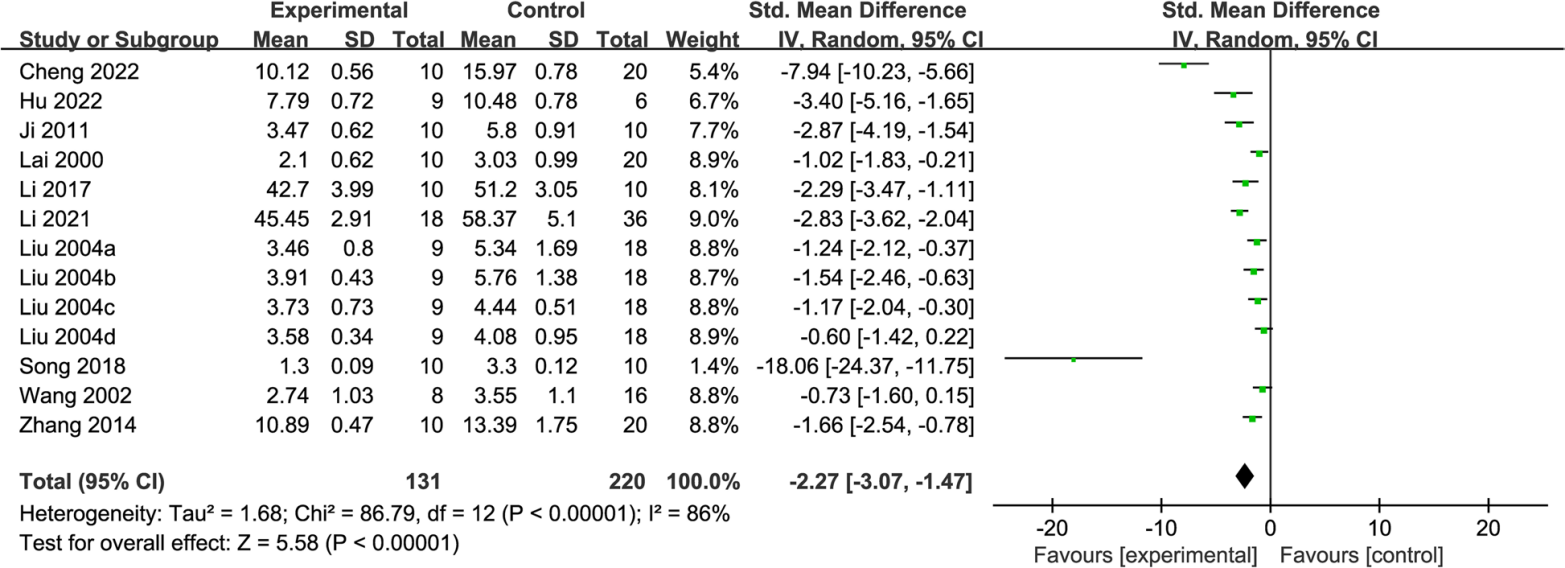
Forest plot of MDA for acupuncture vs. control

Ten studies [26, 31, 35–37, 41, 42, 44, 45, 48] reported MDA levels in a total of 354 rats. The meta-analysis of these studies showed that acupuncture could substantially reduce the content of MDA compared with controls (SMD = -2.27, 95% CI: -3.07, -1.47, *P* < 0.00001, *I*^2^ = 86%) (Fig. 4).

Subgroup analyses were performed based on acupuncture stimulation types, modelling methods and treatment duration. The results showed that EA (SMD = -8.31, 95% CI: -15.50, -1.12) and MA (SMD = -1.72, 95% CI: -2.27, -1.16) were superior to the control group in reducing MDA content. The treatment durations of ≤ 15 days (SMD = -3.55, 95% CI: -5.60, -1.50) and > 15 days (SMD = -1.70, 95% CI: -2.49, -0.92) showed significantly lower MDA content relative to the control. Significant differences were detected between acupuncture and the control in each modelling method (AL [SMD = -4.55, 95% CI: -7.18, -1.91], AC [SMD = -18.06, 95% CI: -24.37, -11.75], 4-VO [SMD = -1.62, 95% CI: -2.55, -0.69], and TM [SMD = -1.22, 95% CI: -1.61, -0.83]). the heterogeneity was slightly reduced in the MA subgroup (*I*^2^ = 69%), and there was no evidence of heterogeneity in TM (*I*^2^ = 0%), indicating that the modelling methods explained part of the heterogeneity (Additional file 3).

#### NO

**Fig. 5:**
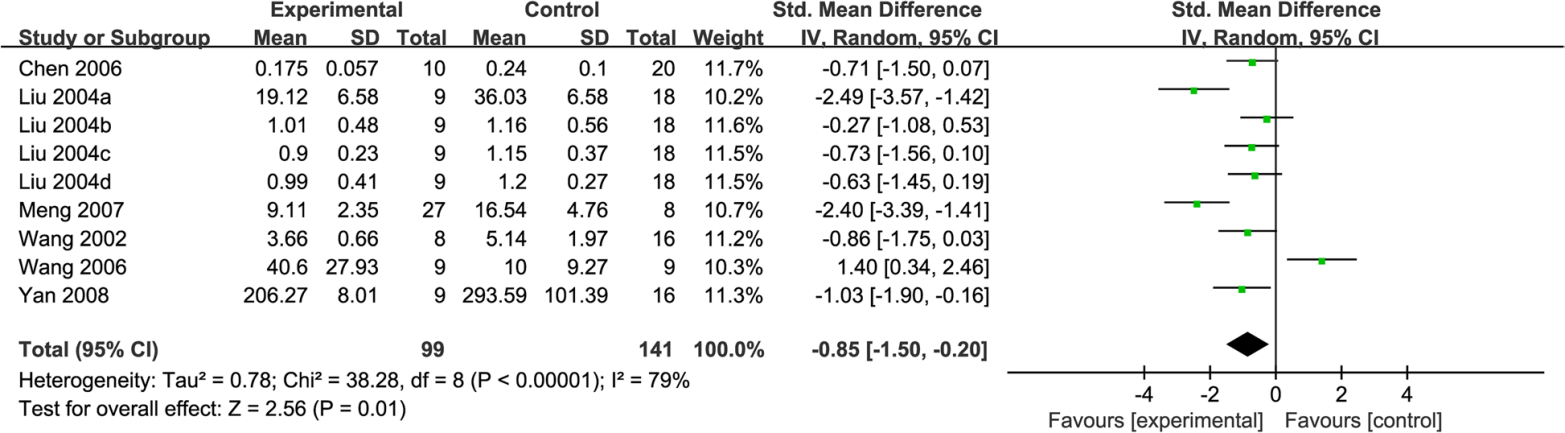
Forest plot of NO for acupuncture vs. control

Six studies [40, 43, 44, 46–48] reported levels of NO, with 240 rats in all. The overall analysis revealed a significant decrease in the content of NO after acupuncture (SMD = -0.85, 95% CI: -1.50, -0.20, *P* = 0.01, *I*^2^ = 79%) (Fig. 5).

After subgroup analyses, statistical differences were found in decreasing NO content in studies using MA stimulation (SMD = -0.97, 95% CI: -1.82, -0.13), 4-VO modelling method (SMD = -0.86, 95% CI: -1.34, -0.37), TM method (SMD = -0.97, 95% CI: -1.82, -0.13), or treatment duration ≤ 15 days (SMD = -1.34, 95% CI: -2.31, -0.38) when acupuncture was compared to the control, whereas the experiments adopting EA stimulation, AL modelling method, and treatment duration > 15 days showed no significant effects (*P* = 0.17, *P* = 0.79, *P* = 0.17, respectively). No heterogeneity (*I*^2^ = 0%) was observed in the 4-VO modelling method (Additional file 3).

#### NOS

**Fig. 6:**
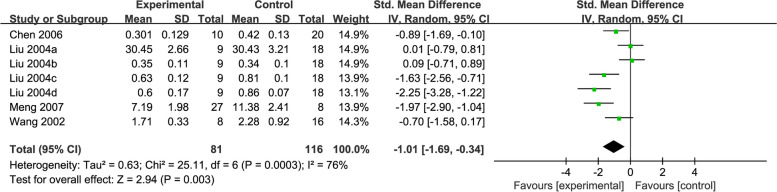
Forest plot of NOS for acupuncture vs. control

Four studies [43, 44, 47, 48] reported on NOS, with 197 rats in total. Pooled results showed that the activity of NOS was significantly lower in the acupuncture group relative to the control group (SMD = -1.01, 95% CI: -1.69, -0.34, *P* = 0.003,* I*^2^ = 76%) (Fig. 6).

The results of the subgroup analysis showed that EA (SMD = -1.17, 95% CI: -1.91, -0.43), AL modelling method (SMD = -1.97, 95% CI: -2.90, -1.04), 4-VO method (SMD = -0.81, 95% CI: -1.40, -0.22), or treatment duration ≤ 15 days (SMD = -1.40, 95% CI: -2.46, -0.35) resulted in lower NOS activity than control. However, no difference was found in studies using MA (*P* = 0.11), TM methods (*P* = 0.11), or treatment duration > 15 days (*P* = 0.05). There was no heterogeneity in the subgroup of the 4-VO method (*I*^2^ = 0%), and lower heterogeneity was observed in the EA (*I*^2^ = 54%) and treatment duration ≤ 15 days (*I*^2^ = 66%) subgroups. which suggested that these may be a source of heterogeneity (Additional file 3).

#### SOD

**Fig. 7:**
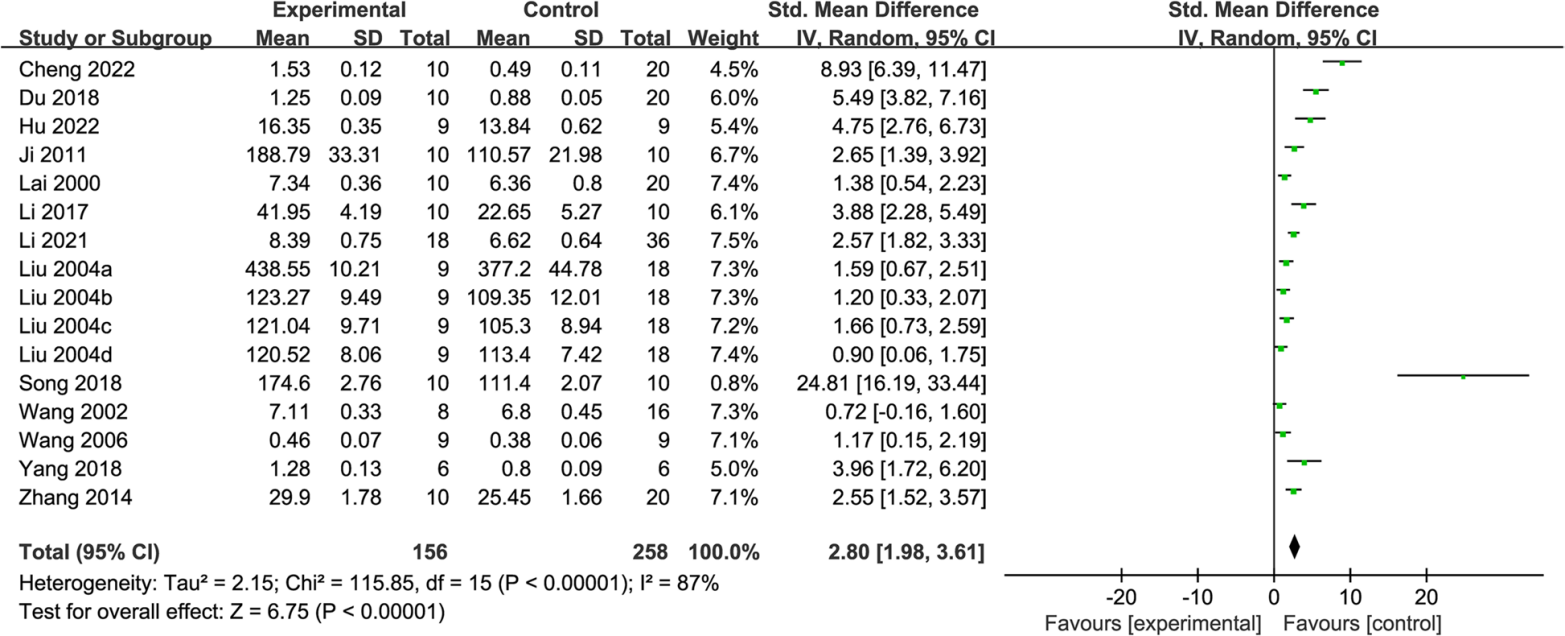
Forest plot of SOD for acupuncture vs. control

Thirteen studies [25, 26, 31, 33, 35–37, 40–42, 44, 45, 48] reported about changes in SOD, evaluating 414 rats in total. The meta-analysis showed that acupuncture was more effective in increasing SOD activity in the intervention group than the control (SMD = 2.80, 95% CI: 1.98, 3.61, *P* < 0.00001, *I*^2^ = 87%) (Fig. 7).

According to the subgroup analyses based on acupuncture stimulation types, modelling methods, and treatment duration, statistically significant differences were revealed in all subgroups, including EA (SMD = 6.00, 95% CI: 2.40, 9.60), MA (SMD = 2.43, 95% CI: 1.71, 3.15), AL method (SMD = 4.27, 95% CI: 2.46, 6.08), AC method (SMD = 24.8, 95% CI: 16.19, 33.44), 4-VO method (SMD = 2.02, 95% CI: 0.81, 3.22), TM method (SMD = 1.53, 95% CI: 1.01, 2.06), treatment duration ≤ 15 days (SMD = 4.28, 95% CI: 2.54, 6.03), and treatment duration > 15 days (SMD = 1.94, 95% CI: 1.12, 2.75) subgroups. Reduced heterogeneity was only detected in the subgroup of TM modelling methods (*I*^2^ = 39%) (Additional file 3).

### GSH-Px

**Fig. 8:**
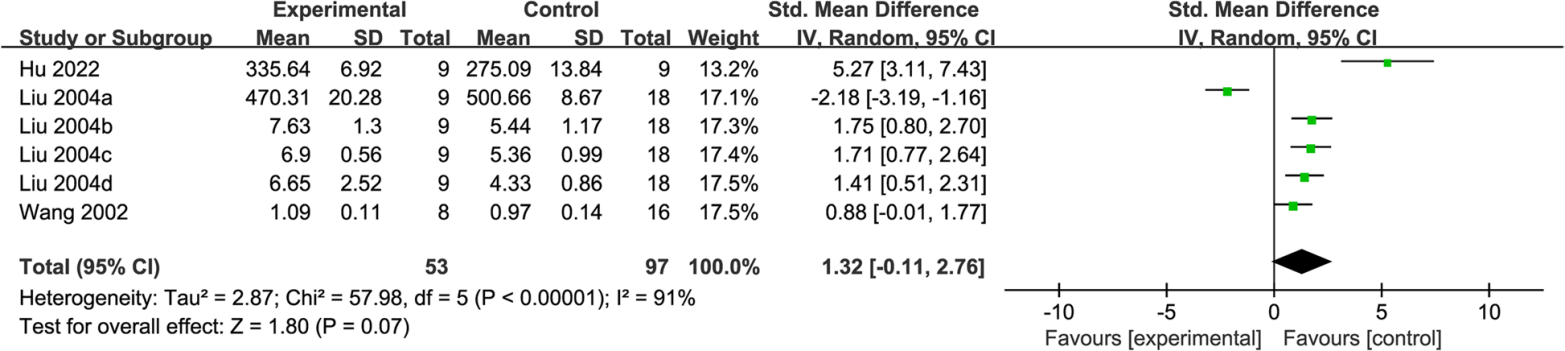
Forest plot of GSH-Px for acupuncture vs. control

GSH-Px was reported in three studies [31, 44, 48] including 150 rats. Pooled data indicated that acupuncture did not show significant effect in elevating GSH-Px activity when compared with the control (SMD = 1.32, 95% CI: -0.11, 2.76, *P* = 0.07, *I*^2^ = 91%) (Fig. 8).

Subgroup analysis results based on different acupuncture types, modelling methods, and treatment duration suggested that there were significant differences in comparisons of AL method (SMD = 5.27, 95% CI: -3.11, 7.43), treatment duration ≤ 15 days (SMD = 5.27, 95% CI: 3.11, 7.43) and control, no significant difference was detected in other subgroups. Substantial heterogeneity remained in the subgroup analyses (*I*^2^ = 93%, *I*^2^ = 93%, *I*^2^ = 91%) (Additional file 3).

#### CAT

**Fig. 9:**
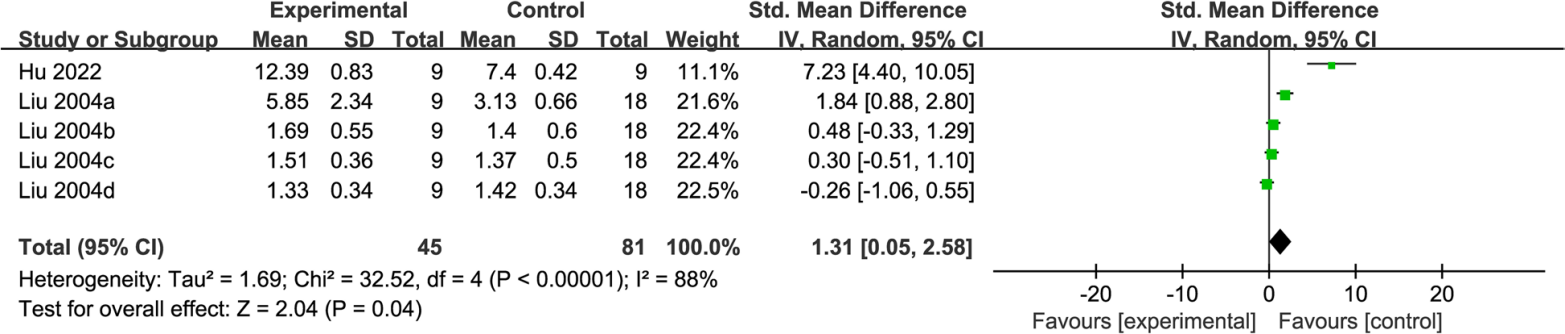
Forest plot of CAT for acupuncture vs. control

Two studies [31, 44] reported about CAT, with 126 rats totally. Meta-analysis results showed that the acupuncture was better than the controls (SMD = 1.31, 95% CI: 0.05, 2.58, *P* = 0.04, *I*^2^ = 88%) in general (Fig. 9).

The subgroup analysis based on modelling methods and treatment duration indicated no statistical difference in studies utilizing the TM method (SMD = 0.56, 95% CI: -0.25, 1.37), and treatment duration > 15 days (SMD = 0.56, 95% CI: -0.25, 1.37), and the heterogeneity did not decrease significantly (*I*^2^ = 73%, *I*^2^ = 73%) (Additional file 3).

### Morris water maze test—escape latency

**Fig. 10:**
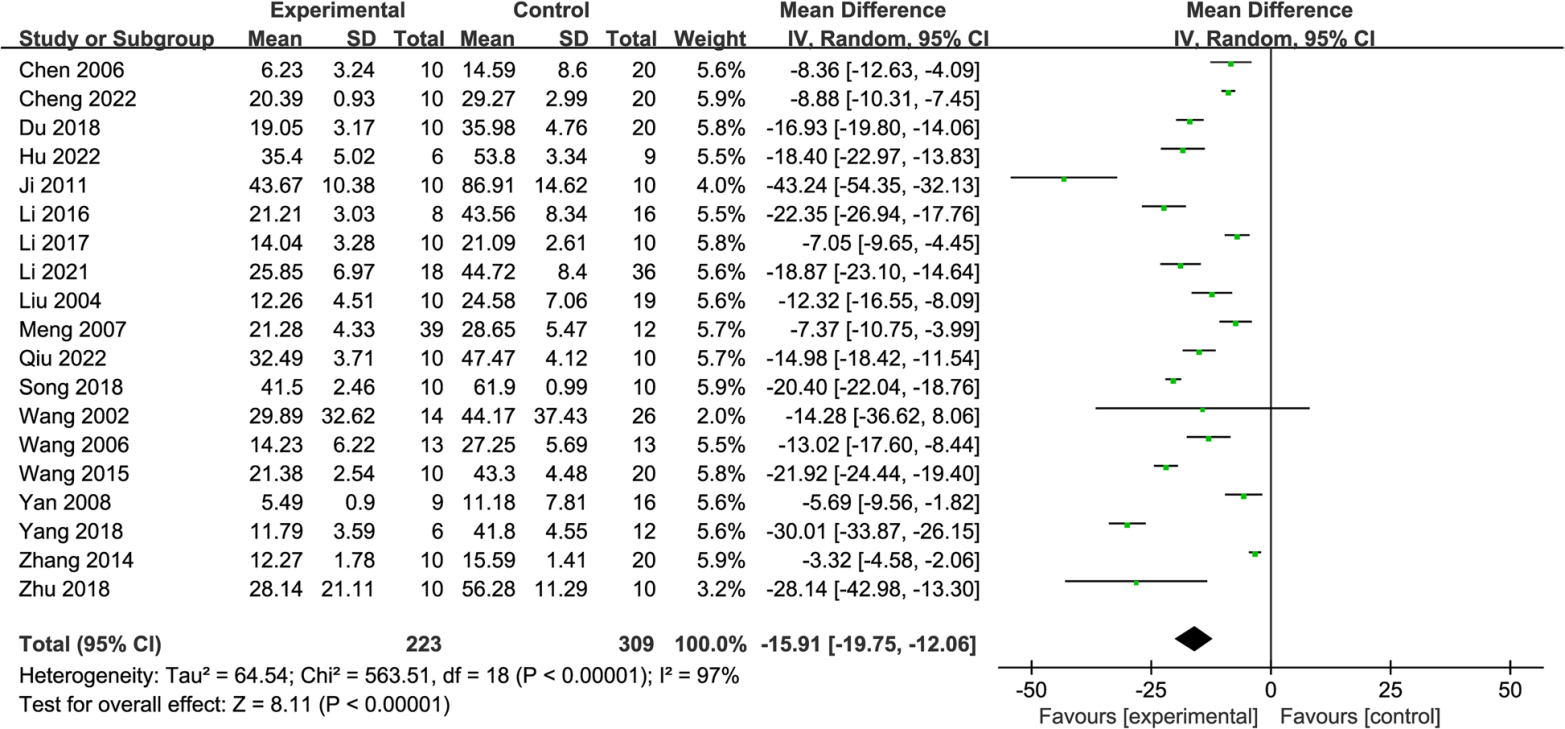
Forest plot of escape latency for acupuncture vs. control

Nineteen studies [24–26, 30–37, 40, 42–48] including 535 rats evaluated escape latency as an outcome. The meta-analysis indicated that rats that underwent acupuncture indeed performed better with shorter escape latency, whereas the control group did not (MD = -15.91, 95% CI: -19.75, -12.06, *P* < 0.00001, *I*^2^ = 97%) (Fig. 10).

Subgroup analyses were performed on the basis of different acupuncture stimulation types, modelling methods, and treatment duration, statistically significant effects were found in all subgroups but TM method (SMD = -7.58, 95% CI: -16.39, 1.22), with substantial heterogeneity remained (Additional file 3).

### Morris water maze test—platform crossing number

**Fig. 11:**
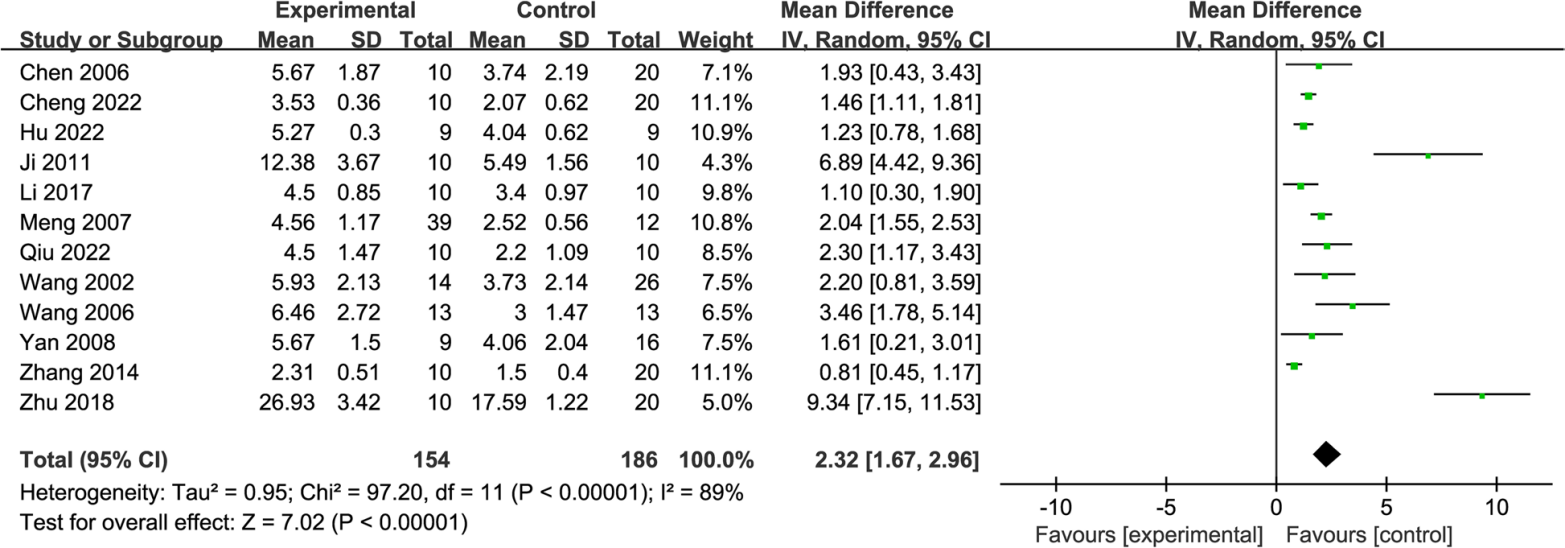
Forest plot of platform crossing number for acupuncture vs. control

The number of platform crossing was measured in 340 rats in 12 studies [24, 31, 32, 35–37, 40, 43, 45–48]. The meta-analysis displayed that the platform crossing number in the acupuncture group was significantly greater than that in the control group (MD = 2.32, 95% CI: 1.67, 2.96, *P* < 0.00001, *I*^2^ = 89%) (Fig. 11).

As the results of the subgroup analysis showed, acupuncture manifested greater advantages in platform crossing number than the control in each subgroup according to acupuncture stimulation types, modelling methods, and treatment duration, and the heterogeneity reduced in the subgroups of EA (*I*^2^ = 55%) and treatment duration ≤ 15 days (*I*^2^ = 37%) (Additional file 3).

### Morris water maze test—duration in the platform quadrant

**Fig. 12:**
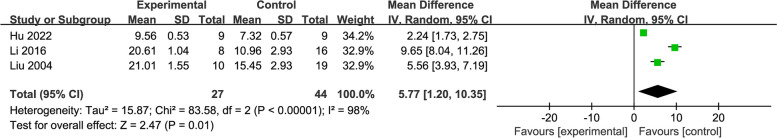
Forest plot of the duration in the platform quadrant for acupuncture vs. control

Three studies [30, 31, 44] including 71 rats used the duration in the platform quadrant as an outcome. The meta-analysis showed that the time in the platform quadrant was significantly higher in the acupuncture group compared with the control group (MD = 5.77, 95% CI: 1.20, 10.35, *P* = 0.01, *I*^2^ = 98%) (Fig. 12).

Subgroup analyses on the basis of different modelling methods and treatment duration showed that acupuncture was not superior to the control in studies using the AL modelling method (MD = 5.90, 95% CI: -1.36, 13.17) or treatment duration ≤ 15 days (MD = 5.90, 95% CI: -1.36, 13.17). Substantial heterogeneities were noted in the above subgroups (*I*^2^ = 99%; *I*^2^ = 99%) (Additional file 3).

### Morris water maze test – swimming speed

**Fig. 13:**
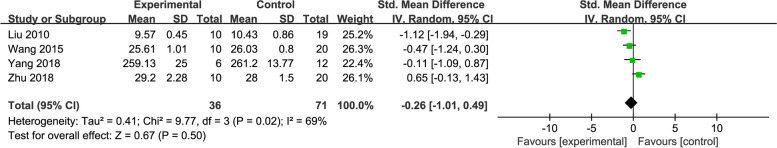
Forest plot of swimming speed for acupuncture vs. control

Five studies [24, 25, 34, 38, 39] with 155 rats showed no significantly differences in swimming speed between acupuncture and control (SMD = -0.26, 95% CI: -1.01, 0.49, *P* = 0.50, *I*^2^ = 69%) (Fig. 13).

The subgroup analyses based on modelling methods and treatment durations indicated that acupuncture had no advantage in improving the swimming speed in the subgroup of the AL modelling method (SMD = 0.03, 95% CI: -0.66, 0.73). Neither the ≤ 15 days (MD = 5.90, 95% CI: -1.36, 13.17) nor the > 15 days (SMD = -0.33, 95% CI: -0.94, 0.27) treatment duration exert a significant effect. The AL modelling method had slightly reduced the heterogeneity (*I*^2^ = 52%), and there was no evidence of heterogeneity in the subgroup of treatment duration ≤ 15 days (*I*^2^ = 0%) (Additional file 3).

### Publication bias

**Fig. 14:**
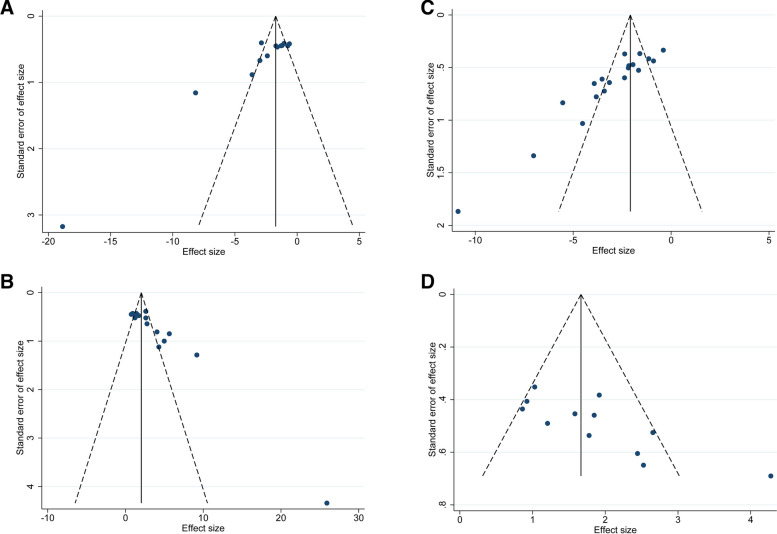
**A** Funnel plot of acupuncture vs. control on MDA **B** Funnel plot of acupuncture vs. control on SOD **C** Funnel plot of acupuncture vs. control on escape latency **D** Funnel plot of acupuncture vs. control on platform crossing number

Publication bias tests were conducted on the MDA, SOD, escape latency, and platform crossing number data. The funnel plot appeared to be asymmetrically distributed on both sides of the midline (Fig. 14. A-D), suggesting the potential presence of publication bias.

### Sensitivity analyses

Sensitivity analyses were performed based on the outcomes of MDA, SOD, escape latency, and platform crossing number by excluding studies with a high ROB. The combined estimates on the remaining studies were basically consistent with the original total effects, indicating that the results of the meta-analysis were stable. No obvious decrease in heterogeneity was found. A sensitivity analysis was not conducted for the other outcomes, only a few studies were included (< 10). The sensitivity analysis results are shown in Additional file 4.

## Discussion

Accumulated evidence [49, 50] suggests a strong correlation between oxidative stress and VaD. Over the past years, numerous clinical trials [51–53] have reported the therapeutic effect of acupuncture on VaD, and basic experiments [24, 25] have implied that its efficacy is linked to its antioxidant function. However, no study has systematically analysed the effect of acupuncture on oxidative stress indicators as its underlying mechanism.

### Role of oxidative stress

Oxidative stress reflects a pathological imbalance between ROS formation and antioxidant activity [14]. Studies have shown that oxidative stress is closely related to blood brain barrier disruption and typical white matter lesions in VaD [54]. ROS are strong contributors to cerebrovascular injury and dementia [50]. When ROS levels reach a critical value, they activate anomalous signalling mechanisms that can result in lipid peroxidation, DNA damage, and protein modifications [55, 56] and exacerbates cerebral ischemia and hypoxia, which are the common features in VaD. MDA is a product of lipid peroxidation and therefore can be used as an indirect measure of cumulative lipid peroxidation [57]. A high level of MDA can overwhelm the antioxidant defence system in vivo and induce cell apoptosis or other pathological reactions. Excessive ROS includes NO. The NO produced by endothelial NOS plays a neuroprotective role in cerebral ischemic injury, while NO released by excessive activation of neuronal NOS and later, inducible NOS contributes to brain damage [58]; most of the included studies in this review measured the latter. A variety of antioxidants, such as SOD, GSH-Px, and CAT, counterbalance the possible deleterious effects and protect from brain injury by eliminating excessive free radicals [59]. SOD, recognized as the first line of defence against oxygen free radicals, has dual roles in limiting ROS toxicity and regulating redox signalling [60]. GSH-Px is a peroxidase that catalyses the production of oxidized glutathione, which scavenges ROS and lipid peroxides [61]. CAT is the marker enzyme of the peroxisome and destroys cellular hydrogen peroxide to produce oxygen and water [62]. Thus, weakening pro-oxidants and enhancing antioxidant defences are important strategies for decreasing oxidative damage.

### Summary of the main findings

This systematic review summarizes the results of 22 studies (747 animals in total) that report the influence of acupuncture on oxidative stress indicators and cognitive function in animal models of VaD. The results of the present meta-analysis indicated that, overall, acupuncture significantly inhibits the expression of pro-oxidants (ROS, MDA, NO, and NOS), promotes the expression of antioxidants (SOD and CAT), and improves behavioural abilities (escape latency, duration in platform quadrant, and platform crossing number) in animal models of VaD. The findings suggest that acupuncture may improve cognitive function by reducing oxidative stress in pre-clinical models of VaD. For GSH-Px and swimming speed, there were no differences between acupuncture and control, but the reason may be that the number of included studies was small (≤ 5). Owing to the substantial heterogeneities, subgroup analyses were conducted to explore the source of heterogeneities. Significant effects were found in most subgroups classified by acupuncture stimulation types, modelling methods, and treatment duration. Additionally, heterogeneity was partially well-explained, which indicates that the effects of acupuncture on VaD animals and homogeneity between studies were influenced by the above variables to some extent.

### Underlying mechanisms of acupuncture

The mechanisms through which acupuncture inhibits oxidative stress in VaD included: (1) Inhibition of nicotinamide adenine dinucleotide phosphate (NADPH) oxidase (NOX): NOX is a major ROS-producing enzyme [63], which is activated under cerebral hypoperfusion, causing the oxidative stress and consequential neuronal death and cognitive impairment involved in VaD [64]. Studies found that acupuncture protects cognition in rats with cerebral ischemia by inhibiting NOX-mediated oxidative stress [65]. (2) Regulation of mitochondrial dysfunction: mitochondria have crucial functions in the regulation of ROS production and respiratory chain. In the brain, oxidative damage decreases the enzymatic activity of the respiratory chain, resulting in mitochondrial dysfunction [66]. Studies suggested that acupuncture could ameliorate brain neuronal damage in VaD rats by reversing hippocampal mitochondrial dysfunction and maintaining mitochondrial homeostasis [30, 37]. (3) Modulation of proteins and enzymes: The thioredoxin (Trx) system is composed of potent protein disulphide reductases that play a critical role in controlling the cellular redox environment and protect tissues and cells against oxidative stress [67], while thioredoxin-interacting protein (TXNIP) oxidizes Trx, mediating an immune response to ROS overproduction, thereby enhancing oxidative stress. Research has indicated that acupuncture improved VaD through antioxidative mechanisms that involved the upregulation of Trx-1/TrxR-1 and downregulation of TXNIP levels [24, 33]. (4) The nuclear factor erythroid 2-like 2 (Nrf2), a transcription factor, is a critical regulator of the antioxidant response system that controls the expression of a wide range of antioxidant genes [68]. Evidence has demonstrated that acupuncture protects cerebral function in VaD models via Nrf2 activation [34].

### Strengths and limitations

This study systematically evaluated the efficacy of acupuncture on VaD for the first time by analysing oxidative stress indicators, and the findings can provide guidance for the design, conduct, and analysis of future basic and clinical research on VaD; This review was pre-registered in the PROSPERO and all stages were reported in compliance with the PRISMA recommendations. The SYRCLE tool was used to assess ROB of included studies; the study focused on multiple oxidative stress biomarkers (4 pro-oxidants and 3 antioxidants) assayed in current research and presents an overall profile of oxidative stress. The results of behavioural tests have also been analysed to confirm the relationship of cognitive improvement with changes in oxidative stress.

This review has some inevitable limitations. First, the databases searched were limited to English and Chinese databases, some relevant literature in other languages may have been omitted. Second, the methodological quality of most studies was uncertain or flawed, mainly due to unclear baseline comparability, sequence generation and random outcome selection, lack of allocation concealment, and blinding outcome evaluation. which has been identified as major challenges in preclinical research [69]. Third, there was considerable heterogeneity among the included studies, although subgroup analyses explained part of these discrepancies, this limits the certainty and comparability of our findings.

### Implications for research

Based on this study’s findings, there are still some knowledge gaps between clinical and basic research that need to be addressed in the future. First, only a few studies have explored the impact of acupuncture on oxidative stress in patients with VaD. More clinical trials should be conducted to further validate the results of basic research and translate them into clinical evidence. Second, the adverse reactions of acupuncture, such as bleeding, infection, and inflammation of the acupoint, should be reported to evaluate the safety and tolerability of acupuncture in the treatment of VaD. Third, it is necessary to establish a standard for oxidative stress analysis, e.g., a complete oxidative stress profile or an analysis of multiple biomarkers, to determine the threshold associated with VaD. Fourth, the effects of acupuncture on NOS, GSH-Px and CAT in animal models of VaD still need to be further investigated to obtain more credible results. In addition, methodological quality problems should be avoided in future RCTs to gain more accurate results in this promising field. As for the high heterogeneity between the studies, a standard acupuncture scheme and uniform animal modelling methods should be established to minimize the heterogeneity.

## Conclusions

Acupuncture might ameliorate cognitive impairment in VaD by regulating oxidative stress indicators. However, firm conclusions cannot be drawn due to the poor methodological quality. Therefore, future research following rigorous standards are still needed to gain more validated information on the effects of acupuncture on oxidative stress in VaD.

### Supplementary Information

**Additional file 1. **PRISMA checklist.

**Additional file 2. **Search strategies of each database.

**Additional file 3. **The results of subgroup analyses for each outcome.

**Additional file 4. **Results of the sensitivity analyses.
