# Supplementary material for: The effect of acupuncture on oxidative stress in animal models of vascular dementia: a systematic review and meta-analysis
Source: Syst Rev. 2024 Feb 8;13:59. doi: 10.1186/s13643-024-02463-x (PMC10851587; doi:10.1186/s13643-024-02463-x)
Supplement: Supplementary file 2 — Additional file 2. Search strategies of each database. [file 13643_2024_2463_MOESM2_ESM.docx]

**Additional file 2:** **Search strategies of each database**

**PubMed**

#1 "Dementia, Vascular"[Mesh] OR "Dementia, Multi-Infarct"[Mesh]

#2 "dementia, vascular"[Title/Abstract] OR "vascular dementia*"[Title/Abstract] OR "dementia, subcortical vascular"[Title/Abstract] OR "arteriosclerotic dementia*"[Title/Abstract] OR "dementia, arteriosclerotic"[Title/Abstract] OR "Binswanger disease"[Title/Abstract] OR "disease, Binswanger"[Title/Abstract] OR "multi-infarct dementia"[Title/Abstract] OR "multiinfarct dementia"[Title/Abstract] OR "infarct dementia"[Title/Abstract] OR "post-stroke dementia"[Title/Abstract] OR "poststroke dementia"[Title/Abstract] OR "stroke dementia"[Title/Abstract] OR "vascular cognitive impairment"[Title/Abstract] OR "VaD"[Title/Abstract] OR "VD"[Title/Abstract]

#3 #1 OR #2

#4 "Acupuncture therapy"[Mesh] OR "Electroacupuncture"[Mesh] OR "Acupuncture"[Mesh]

#5 "acupuncture"[Title/Abstract] OR "electroacupuncture"[Title/Abstract] OR "acupun*" [Title/Abstract] OR "needle*" OR "meridian*"[Title/Abstract] OR "acupoint*"[Title/Abstract] OR "electro-acupuncture"[Title/Abstract] OR "manual-acupuncture"[Title/Abstract] OR "pharmacopuncture"[Title/Abstract] OR "pharmacoacupuncture"[Title/Abstract]

#6 #4 OR #5

#7 "Models, Animal"[MeSH] OR "Animal Experimentation"[MeSH]

#8 "rat"[Title/Abstract] OR "rats"[Title/Abstract] OR "mouse"[Title/Abstract] OR "mice"[Title/Abstract] OR "rabbit"[Title/Abstract] OR "rabbits"[Title/Abstract] OR "dog"[Title/Abstract] OR "pig"[Title/Abstract] OR "animal"[Title/Abstract] OR "animals"[Title/Abstract] OR "experiment*"[Title/Abstract]

#9 #7 OR #8

#10 #3 AND #6 AND #9

**EMBASE**

#1 'dementia'/exp/mj

#2 'dementia, vascular':ti,ab,kw OR 'vascular dementia*':ti,ab,kw OR 'dementia, subcortical vascular':ti,ab,kw OR 'arteriosclerotic dementia*':ti,ab,kw OR 'dementia, arteriosclerotic':ti,ab,kw OR 'Binswanger disease':ti,ab,kw OR 'disease, Binswanger':ti,ab,kw OR 'multi-infarct dementia':ti,ab,kw OR 'multiinfarct dementia':ti,ab,kw OR 'infarct dementia':ti,ab,kw OR 'post-stroke dementia':ti,ab,kw OR 'poststroke dementia':ti,ab,kw OR 'stroke dementia':ti,ab,kw OR 'vascular cognitive impairment':ti,ab,kw OR 'VaD':ti,ab,kw OR 'VD':ti,ab,kw

#3 #1 OR #2

#4 'acupuncture'/exp/mj OR 'electroacupuncture'/exp/mj

#5 'acupuncture':ti,ab,kw OR 'acupun*':ti,ab,kw OR 'needle*':ti,ab,kw OR 'meridian*':ti,ab,kw OR 'acupoint*':ti,ab,kw OR 'electro-acupuncture':ti,ab,kw OR 'electroacupuncture':ti,ab,kw OR 'manual-acupuncture':ti,ab,kw OR 'pharmacopuncture':ti,ab,kw OR 'pharmacoacupuncture':ti,ab,kw

#6 #4 OR #5

#7 'animal model'/exp/mj OR 'animal experimentation'/exp/mj

#8 'rat':ti,ab,kw OR 'rats':ti,ab,kw OR 'mouse':ti,ab,kw OR 'mice':ti,ab,kw OR 'rabbit':ti,ab,kw OR 'rabbits':ti,ab,kw OR 'dog':ti,ab,kw OR 'pig':ti,ab,kw OR 'animal':ti,ab,kw OR 'animals':ti,ab,kw OR 'experiment*':ti,ab,kw

#9 #7 OR #8

#10 #3 AND #6 AND #9

**Web of science**

TS=(‘dementia, vascular’ OR ‘vascular dementia*’ OR ‘dementia, subcortical vascular’ OR ‘arteriosclerotic dementia*’ OR ‘dementia, arteriosclerotic’ OR ‘Binswanger disease’ OR ‘disease, Binswanger’ OR ‘multi-infarct dementia’ OR ‘*infarct dementia’ OR ‘post-stroke dementia’ OR ‘*stroke dementia’ OR ‘vascular cognitive impairment’ OR ‘VaD’ OR ‘VD’) AND TS=(‘acupuncture’ OR ‘electroacupuncture’ OR ‘*acupuncture’ OR ‘acupun*’ OR ‘needle*’ OR ‘meridian*’ OR ‘acupoint*’ OR ‘electro-acupuncture’ OR ‘manual-acupuncture’ OR ‘pharmacopuncture’ OR ‘pharmacoacupuncture’) AND TS=(‘rat’ OR ‘rats’ OR ‘mouse’ OR ‘mice’ OR ‘rabbit’ OR ‘rabbits’ OR ‘dog’ OR ‘pig’ OR ‘animal*’ OR ‘experiment*’)

**Cochrane Library**

#1 Mesh descriptor: [Dementia, Vascular] explode all trees

#2 Mesh descriptor: [Dementia, Multi-Infarct] explode all trees

#3 "dementia, vascular":ti,ab,kw OR "vascular dementia":ti,ab,kw OR "dementia, subcortical vascular":ti,ab,kw OR "arteriosclerotic dementia":ti,ab,kw OR "dementia, arteriosclerotic:ti,ab,kw OR "Binswanger disease":ti,ab,kw OR "disease, Binswanger":ti,ab,kw OR "multi-infarct dementia":ti,ab,kw OR "multiinfarct dementia":ti,ab,kw OR "infarct dementia":ti,ab,kw OR "post-stroke dementia":ti,ab,kw OR "stroke dementia":ti,ab,kw OR "vascular cognitive impairment":ti,ab,kw OR VaD:ti,ab,kw OR VD:ti,ab,kw

#4 #1 OR #2 OR #3

#5 Mesh descriptor: [Acupuncture] explode all trees;

#6 Mesh descriptor: [Acupuncture therapy] explode all trees

#7 acupuncture:ti,ab,kw OR *acupuncture:ti,ab,kw OR acupun*:ti,ab,kw OR needle*:ti,ab,kw OR meridian*:ti,ab,kw OR acupoint*:ti,ab,kw OR electro-acupuncture:ti,ab,kw OR electroacupuncture:ti,ab,kw OR manual-acupuncture:ti,ab,kw OR pharmacopuncture:ti,ab,kw

#8 #5 OR #6 OR #7

#9 Mesh descriptor: [Models, Animal] explode all trees

#10 Mesh descriptor: [Animal Experimentation] explode all trees

#11 rat:ti,ab,kw OR rats:ti,ab,kw OR mouse:ti,ab,kw OR mice:ti,ab,kw OR rabbit:ti,ab,kw OR rabbits:ti,ab,kw OR dog:ti,ab,kw OR pig:ti,ab,kw OR animal:ti,ab,kw OR animals:ti,ab,kw OR experiment*:ti,ab,kw

#12 #9 OR #10 OR #11

#13 #4 AND #8 AND #12

**CNKI**

(TKA=(‘针刺’+‘针灸’+‘体针’+‘手针’+‘电针’+‘头皮针’+‘头针’+‘耳针’+‘穴位’+‘火针’+‘腹针’+‘浮针’+‘眼针’+‘舌针’+‘腕踝针’+‘干针’) OR SU=(‘针刺疗法’+‘针刺’+‘针灸疗法’)) AND (TKA=(‘血管性痴呆’+‘动脉硬化性痴呆’+‘梗塞性痴呆’+‘梗死性痴呆’+‘卒中后痴呆’+‘宾斯旺格病’+‘血管性认知障碍’+‘血管性认知损害’+‘血管性认知损伤’) OR SU=(‘痴呆, 血管性’+‘痴呆, 多发性梗死性’)) AND (TKA=(‘动物’+‘鼠’+‘兔’+‘狗’+‘猪’+ ‘基础研究’) OR SU=(‘动物模型’+ ‘动物实验’))

**WF**

(题名或关键词:(血管性痴呆 OR 动脉硬化性痴呆 OR 梗塞性痴呆OR 梗死性痴呆 OR卒中后痴呆OR 宾斯旺格病OR血管性认知障碍 OR 血管性认知损害OR 血管性认知损伤) OR 主题:(痴呆，血管性 OR 痴呆, 多发性梗死性)) and (题名或关键词:(针刺 OR 针灸 OR 体针 OR 手针 OR 电针 OR 头皮针 OR 头针 OR 耳针 OR 穴位 OR 火针 OR 腹针 OR 浮针 OR 眼针 OR 舌针 OR 腕踝针 OR 干针) OR 主题:(针刺疗法 OR 针灸疗法 OR 针刺)) and (题名或关键词:(动物 OR 鼠 OR 兔 OR 狗 OR 猪OR 基础研究) OR 主题:(动物实验 OR 动物模型))

**Chongqing VIP**

M=(血管性痴呆 OR 动脉硬化性痴呆 OR 梗塞性痴呆OR 梗死性痴呆 OR卒中后痴呆 OR 宾斯旺格病 OR血管性认知障碍 OR 血管性认知损害OR 血管性认知损伤) and M=(针刺 OR 针灸 OR 体针 OR 手针 OR 电针 OR 头皮针 OR 头针 OR 耳针 OR 穴位 OR 火针 OR 腹针 OR 浮针 OR 眼针 OR 舌针 OR 腕踝针 OR 干针) and R=(动物 OR 鼠 OR 兔 OR 狗 OR 猪 OR 基础研究)

**CBM**

1 "痴呆, 血管性" [加权:扩展] OR "痴呆, 多发性梗死性"[加权:扩展]

2 "血管性痴呆"[常用字段:智能] OR "动脉硬化性痴呆"[常用字段:智能] OR "梗塞性痴呆"[常用字段:智能] OR "梗死性痴呆"[常用字段:智能] OR "卒中后痴呆"[常用字段:智能] OR "宾斯旺格病"[常用字段:智能] OR "血管性认知障碍" OR "血管性认知损害"[常用字段:智能] OR "血管性认知损伤"[常用字段:智能]

3 1 OR 2

4 "针刺疗法"[加权:扩展] OR "针刺"[加权:扩展] OR "针灸疗法"[加权:扩展]

5 "针刺"[常用字段:智能] OR "针灸"[常用字段:智能] OR "体针"[常用字段:智能] OR "手针"[常用字段:智能] OR "电针"[常用字段:智能] OR "头皮针"[常用字段:智能] OR "头针"[常用字段:智能] OR "耳针"[常用字段:智能] OR "穴位"[常用字段:智能] OR "火针"[常用字段:智能] OR "腹针"[常用字段:智能] OR "浮针"[常用字段:智能] OR "舌针"[常用字段:智能] OR "腕踝针"[常用字段:智能] OR "眼针"[常用字段:智能] OR "干针"[常用字段:智能]

6 4 OR 5

7 "动物模型" [加权:扩展] OR "动物实验"[加权:扩展]

8 "动物"[常用字段:智能] OR "鼠"[常用字段:智能] OR "兔"[常用字段:智能] OR "狗"[常用字段:智能] OR "猪"[常用字段:智能] OR "基础研究"[常用字段:智能]

9 7 OR 8

10 3 AND 6 AND 9
