# Supplementary material for: The effect of acupuncture on oxidative stress in animal models of vascular dementia: a systematic review and meta-analysis
Source: Syst Rev. 2024 Feb 8;13:59. doi: 10.1186/s13643-024-02463-x (PMC10851587; doi:10.1186/s13643-024-02463-x)
Supplement: Supplementary file 3 — Additional file 3. The results of subgroup analyses for each outcome. [file 13643_2024_2463_MOESM3_ESM.docx]

**Additional file 3: The results of subgroup analyses for each outcome**

(A). The subgroup analysis for the ROS

| Subgroup | Eligible Studies | Intervention group (n) | Control  group (n) | Standardized Mean Difference [95% CI] | *P* value | Heterogeneity test |
| --- | --- | --- | --- | --- | --- | --- |
| Acupuncture simulation type |  |  |  |  |  |  |
| Manual acupuncture | 7 | 65 | 115 | -3.56 [-5.44, -1.68] | 0.0002 | *P* < 0.00001, *I*^2^ = 91% |
| Electroacupuncture | 1 | 10 | 20 | -9.64 [-12.37, -6.92] | < 0.00001 | NA |
| Treatment duration |  |  |  |  |  |  |
| ≤ 15 days | 7 | 71 | 127 | -4.05 [-6.14, -1.96] | 0.0001 | *P* < 0.00001, *I*^2^= 93% |
| > 15 days | 1 | 4 | 8 | -6.35 [-9.68, -3.03] | 0.0002 | NA |

(B). The subgroup analysis for the MDA

| Subgroup | Eligible Studies | Intervention group (n) | Control  group (n) | Standardized Mean Difference [95% CI] | *P* value | Heterogeneity test |
| --- | --- | --- | --- | --- | --- | --- |
| Acupuncture simulation type |  |  |  |  |  |  |
| Manual acupuncture | 10 | 101 | 170 | -1.72 [-2.27, -1.16] | < 0.00001 | *P* = 0.0006, *I*^2^ = 69% |
| Electroacupuncture | 3 | 30 | 50 | -8.31 [-15.50, -1.12] | 0.02 | *P* < 0.00001, *I*^2^ = 96% |
| Modelling method |  |  |  |  |  |  |
| Artery ligation | 3 | 37 | 62 | -4.55 [-7.18, -1.91] | 0.0007 | *P* = 0.0002, *I*^2^ = 88% |
| Artery clamp | 1 | 10 | 10 | -18.06 [-24.37, -11.75] | < 0.00001 | NA |
| 4-blood vessel blocking | 4 | 38 | 56 | -1.62 [-2.55, -0.69] | 0.0007 | *P* = 0.02, *I*^2^ = 70% |
| Thromboembolus | 5 | 46 | 92 | -1.22 [-1.61, -0.83] | < 0.00001 | *P* = 0.46, *I*^2^ = 0% |
| Treatment duration |  |  |  |  |  |  |
| ≤ 15 days | 4 | 47 | 82 | -3.55 [-5.60, -1.50] | 0.0007 | *P* < 0.00001, *I*^2^ = 92% |
| > 15 days | 9 | 84 | 138 | -1.70 [-2.49, -0.92] | < 0.0001 | *P* < 0.00001, *I*^2^ = 80% |

(C). The subgroup analysis for the NO

| Subgroup | Eligible Studies | Intervention group (n) | Control  group (n) | Standardized Mean Difference [95% CI] | *P* value | Heterogeneity test |
| --- | --- | --- | --- | --- | --- | --- |
| Acupuncture simulation type |  |  |  |  |  |  |
| Manual acupuncture | 4 | 36 | 72 | -0.97 [-1.82, -0.13] | 0.02 | *P* = 0.01, *I*^2^ = 73% |
| Electroacupuncture | 5 | 63 | 69 | -0.73 [-1.79, 0.32] | 0.17 | *P* < 0.0001, *I*^2^ = 85% |
| Modelling method |  |  |  |  |  |  |
| Artery ligation | 2 | 36 | 17 | -0.50 [-4.23, 3.22] | 0.79 | *P* < 0.00001, *I*^2^ = 96% |
| 4-blood vessel blocking | 3 | 27 | 52 | -0.86 [-1.34, -0.37] | 0.0006 | *P* = 0.87, *I*^2^ = 0% |
| Thromboembolus | 4 | 36 | 72 | -0.97 [-1.82, -0.13] | 0.02 | *P* = 0.01, *I*^2^ = 73% |
| Treatment duration |  |  |  |  |  |  |
| ≤ 15 days | 3 | 46 | 44 | -1.34 [-2.31, -0.38] | 0.006 | *P* = 0.03, *I*^2^ = 72% |
| > 15 days | 6 | 53 | 97 | -0.60 [-1.44, 0.25] | 0.17 | *P* < 0.0001, *I*^2^ = 81% |

(D). The subgroup analysis for the NOS

| Subgroup | Eligible Studies | Intervention group (n) | Control  group (n) | Standardized Mean Difference [95% CI] | *P* value | Heterogeneity test |
| --- | --- | --- | --- | --- | --- | --- |
| Acupuncture simulation type |  |  |  |  |  |  |
| Manual acupuncture | 4 | 36 | 72 | -0.91 [-2.03, 0.21] | 0.11 | *P* = 0.0002, *I*^2^ = 84% |
| Electroacupuncture | 3 | 45 | 44 | -1.17 [-1.91, -0.43] | 0.002 | *P* = 0.11, *I*^2^ = 54% |
| Modelling method |  |  |  |  |  |  |
| Artery ligation | 1 | 27 | 8 | -1.97 [-2.90, -1.04] | < 0.0001 | NA |
| 4-blood vessel blocking | 2 | 18 | 36 | -0.81 [-1.40, -0.22] | 0.007 | *P* = 0.75, *I*^2^ = 0% |
| Thromboembolus | 4 | 36 | 72 | -0.91 [-2.03, 0.21] | 0.11 | *P* = 0.0002, *I*^2^ = 84% |
| Treatment duration |  |  |  |  |  |  |
| ≤ 15 days | 2 | 37 | 28 | -1.40 [-2.46, -0.35] | 0.009 | *P* = 0.08, *I*^2^ = 66% |
| > 15 days | 5 | 44 | 88 | -0.86 [-1.73, 0.01] | 0.05 | *P* = 0.0007, *I*^2^ = 79% |

(E). The subgroup analysis for the SOD

| Subgroup | Eligible Studies | Intervention group (n) | Control  group (n) | Standardized Mean Difference [95% CI] | *P* value | Heterogeneity test |
| --- | --- | --- | --- | --- | --- | --- |
| Acupuncture simulation type |  |  |  |  |  |  |
| Manual acupuncture | 12 | 117 | 199 | 2.43 [1.71, 3.15] | < 0.00001 | *P* < 0.00001, *I*^2^ = 80% |
| Electroacupuncture | 4 | 39 | 59 | 6.00 [2.40, 9.60] | 0.001 | *P* < 0.00001, *I*^2^ = 95% |
| Modelling method |  |  |  |  |  |  |
| Artery ligation | 6 | 62 | 100 | 4.27 [2.46, 6.08] | < 0.00001 | *P* < 0.00001, *I*^2^ = 89% |
| Artery clamp | 1 | 10 | 10 | 24.81 [16.19, 33.44] | < 0.00001 | NA |
| 4-blood vessel blocking | 4 | 38 | 56 | 2.02 [0.81, 3.22] | 0.001 | *P* = 0.002, *I*^2^ = 79% |
| Thromboembolus | 5 | 46 | 92 | 1.53 [1.01, 2.06] | < 0.00001 | *P* = 0.16, *I*^2^ = 39% |
| Treatment duration |  |  |  |  |  |  |
| ≤ 15 days | 6 | 63 | 111 | 4.28 [2.54, 6.03] | < 0.00001 | *P* < 0.00001, *I*^2^ = 90% |
| > 15 days | 10 | 93 | 147 | 1.94 [1.12, 2.75] | < 0.00001 | *P* < 0.00001, *I*^2^ = 82% |

(F). The subgroup analysis for the GSH-Px

| Subgroup | Eligible Studies | Intervention group (n) | Control  group (n) | Standardized Mean Difference [95% CI] | *P* value | Heterogeneity test |
| --- | --- | --- | --- | --- | --- | --- |
| Acupuncture simulation type |  |  |  |  |  |  |
| Manual acupuncture | 5 | 45 | 81 | 1.45 [-0.37, 3.27] | 0.12 | *P* < 0.00001, *I*^2^ = 93% |
| Electroacupuncture | 1 | 8 | 16 | 0.88 [-0.01, 1.77] | 0.05 | NA |
| Modelling method |  |  |  |  |  |  |
| Artery ligation | 1 | 9 | 9 | 5.27 [3.11, 7.43] | < 0.00001 | NA |
| 4-blood vessel blocking | 1 | 8 | 16 | 0.88 [-0.01, 1.77] | 0.05 | NA |
| Thromboembolus | 4 | 36 | 72 | 0.68 [-1.10, 2.46] | 0.45 | *P* < 0.00001, *I*^2^ = 93% |
| Treatment duration |  |  |  |  |  |  |
| ≤ 15 days | 1 | 9 | 9 | 5.27 [3.11, 7.43] | < 0.00001 | NA |
| > 15 days | 5 | 44 | 88 | 0.73 [-0.63, 2.09] | 0.30 | *P* < 0.00001, *I*^2^ = 91% |

(G). The subgroup analysis for the CAT

| Subgroup | Eligible Studies | Intervention group (n) | Control  group (n) | Standardized Mean Difference [95% CI] | *P* value | Heterogeneity test |
| --- | --- | --- | --- | --- | --- | --- |
| Modelling method |  |  |  |  |  |  |
| Artery ligation | 1 | 9 | 9 | 7.23 [4.40, 10.05] | < 0.00001 | NA |
| Thromboembolus | 4 | 36 | 72 | 0.56 [-0.25, 1.37] | 0.18 | *P* = 0.01, *I*^2^ = 73% |
| Treatment duration |  |  |  |  |  |  |
| ≤ 15 days | 1 | 9 | 9 | 7.23 [4.40, 10.05] | < 0.00001 | NA |
| > 15 days | 4 | 36 | 72 | 0.56 [-0.25, 1.37] | 0.18 | *P* = 0.01, *I*^2^ = 73% |

(H). The subgroup analysis for the escape latency

| Subgroup | Eligible Studies | Intervention group (n) | Control  group (n) | Mean Difference [95% CI] | *P* value | Heterogeneity test |
| --- | --- | --- | --- | --- | --- | --- |
| Acupuncture simulation type |  |  |  |  |  |  |
| Manual acupuncture | 13 | 131 | 224 | -18.09 [-23.87, -12.31] | < 0.00001 | *P* < 0.00001, *I*^2^ = 97% |
| Electroacupuncture | 6 | 92 | 85 | -12.23 [-17.48, -6.98] | < 0.00001 | *P* < 0.00001, *I*^2^ = 96% |
| Modelling method |  |  |  |  |  |  |
| Artery ligation | 11 | 140 | 178 | -17.73 [-22.18, -13.28] | < 0.00001 | *P* < 0.00001, *I*^2^ = 95% |
| Artery clamp | 1 | 10 | 10 | -20.40 [-22.04, -18.76] | < 0.00001 | NA |
| 4-blood vessel blocking | 5 | 53 | 82 | -13.69 [-21.19, -6.19] | 0.0003 | *P* < 0.00001, *I*^2^ = 90% |
| Thromboembolus | 2 | 20 | 39 | -7.58 [-16.39, 1.22] | 0.09 | *P* < 0.0001, *I*^2^ = 94% |
| Treatment duration |  |  |  |  |  |  |
| ≤ 15 days | 10 | 126 | 181 | -15.84 [-20.69, -10.99] | < 0.00001 | *P* < 0.00001, *I*^2^ = 96% |
| > 15 days | 9 | 87 | 118 | -15.26 [-22.15, -8.37] | < 0.00001 | *P* < 0.00001, *I*^2^ = 98% |

(I). The subgroup analysis for the platform crossing number

| Subgroup | Eligible Studies | Intervention group (n) | Control  group (n) | Standardized Mean Difference [95% CI] | *P* value | Heterogeneity test |
| --- | --- | --- | --- | --- | --- | --- |
| Acupuncture simulation type |  |  |  |  |  |  |
| Manual acupuncture | 7 | 72 | 111 | 2.76 [1.61, 3.91] | < 0.00001 | *P* < 0.00001, *I*^2^ = 93% |
| Electroacupuncture | 5 | 82 | 75 | 1.97 [1.44, 2.49] | < 0.00001 | *P* = 0.06, *I*^2^ = 55% |
| Modelling method |  |  |  |  |  |  |
| Artery ligation | 6 | 91 | 84 | 2.71 [1.76, 3.67] | < 0.00001 | *P* < 0.00001, *I*^2^ = 92% |
| 4-blood vessel blocking | 5 | 53 | 82 | 2.42 [1.05, 3.79] | 0.0005 | *P* = 0.0006, *I*^2^ = 80% |
| Thromboembolus | 1 | 10 | 20 | 0.81 [0.45, 1.17] | < 0.0001 | NA |
| Treatment duration |  |  |  |  |  |  |
| ≤ 15 days | 5 | 77 | 77 | 1.55 [1.31, 1.78] | < 0.00001 | *P* = 0.17, *I*^2^ = 37% |
| > 15 days | 7 | 77 | 109 | 1.35 [1.05, 1.65] | < 0.00001 | *P* < 0.00001, *I*^2^ = 93% |

(J). The subgroup analysis for the duration in platform quadrant

| Subgroup | Eligible Studies | Intervention group (n) | Control  group (n) | Mean Difference [95% CI] | *P* value | Heterogeneity test |
| --- | --- | --- | --- | --- | --- | --- |
| Modelling method |  |  |  |  |  |  |
| Artery ligation | 2 | 17 | 25 | 5.90 [-1.36, 13.17] | 0.11 | *P* < 0.00001, *I*^2^ = 99% |
| Thromboembolus | 1 | 10 | 19 | 5.56 [3.93, 7.19] | < 0.00001 | NA |
| Treatment duration |  |  |  |  |  |  |
| ≤ 15 days | 2 | 17 | 25 | 5.90 [-1.36, 13.17] | 0.11 | *P* < 0.00001, *I*^2^ = 99% |
| > 15 days | 1 | 10 | 19 | 5.56 [3.93, 7.19] | < 0.00001 | NA |

(K). The subgroup analysis for the swimming speed

| Subgroup | Eligible Studies | Intervention group (n) | Control  group (n) | Standardized Mean Difference [95% CI] | *P* value | Heterogeneity test |
| --- | --- | --- | --- | --- | --- | --- |
| Modelling method |  |  |  |  |  |  |
| Artery ligation | 3 | 26 | 52 | 0.03 [-0.66, 0.73] | 0.93 | *P* = 0.13, *I*^2^ = 52% |
| Thromboembolus | 1 | 10 | 19 | -1.12 [-1.94, -0.29] | 0.008 | NA |
| Treatment duration |  |  |  |  |  |  |
| ≤ 15 days | 2 | 16 | 32 | -0.33 [-0.94, 0.27] | 0.28 | *P* = 0.57, *I*^2^ = 0% |
| > 15 days | 2 | 20 | 39 | -0.23 [-1.96, 1.51] | 0.80 | *P* = 0.002, *I*^2^ = 89% |
