# Supplementary material for: The effect of acupuncture on oxidative stress in animal models of vascular dementia: a systematic review and meta-analysis
Source: Syst Rev. 2024 Feb 8;13:59. doi: 10.1186/s13643-024-02463-x (PMC10851587; doi:10.1186/s13643-024-02463-x)

**Additional file 4. Results of the sensitivity analyses**

(A). Sensitivity analysis of acupuncture vs. control for MDA


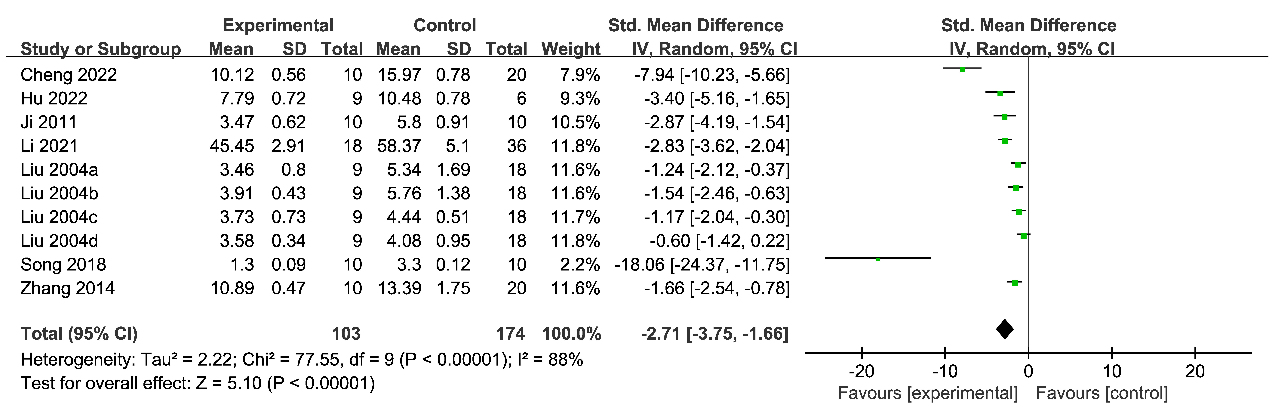


(B). Sensitivity analysis of acupuncture vs. control for SOD


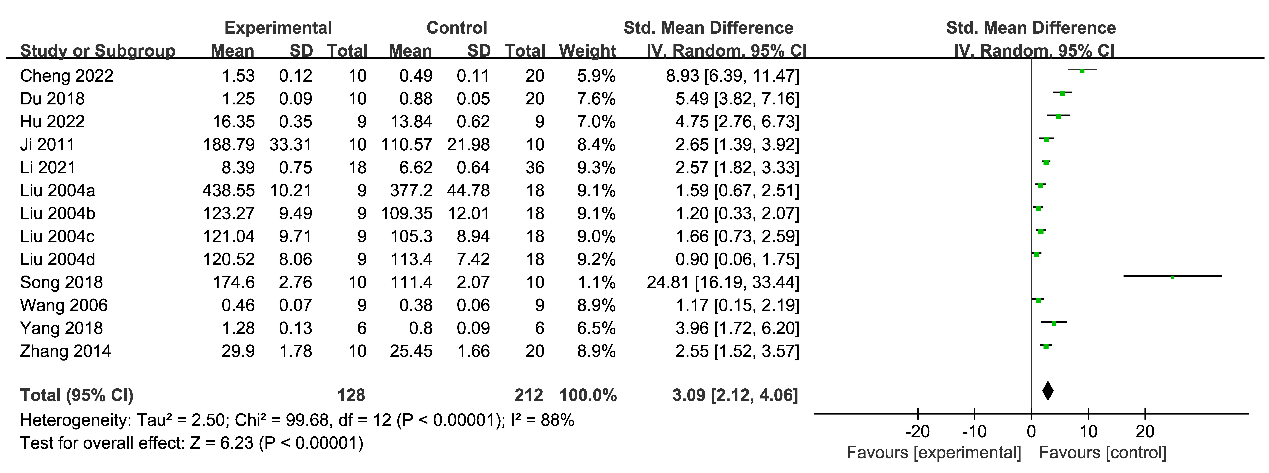


(C). Sensitivity analysis of acupuncture vs. control for escape latency


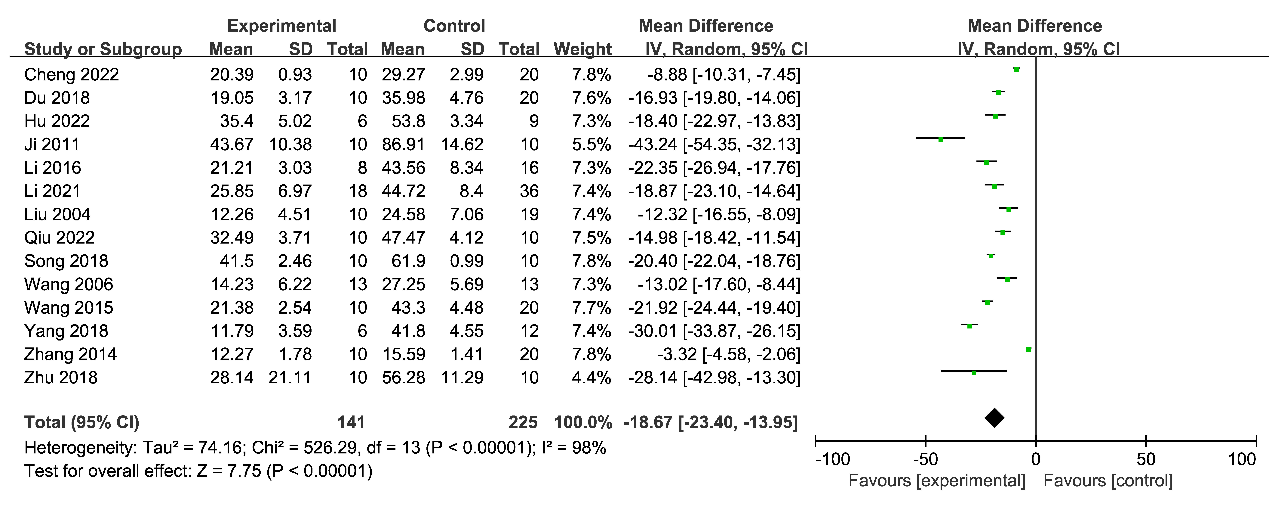


(D). Sensitivity analysis of acupuncture vs. control for platform crossing number


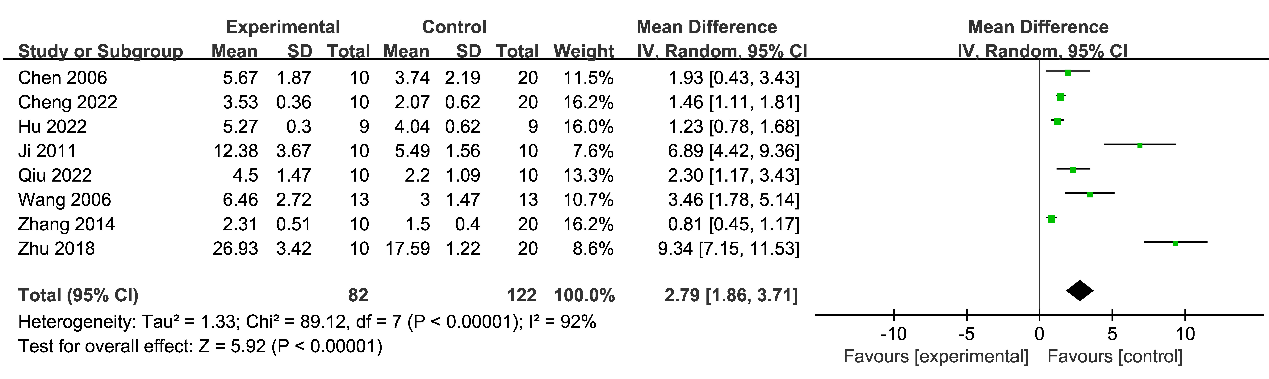

Supplement: Supplementary file 4 — Additional file 4. Results of the sensitivity analyses. [file 13643_2024_2463_MOESM4_ESM.docx]
